# Association Between Temporal Changes in Diet Quality and Concurrent Changes in Dietary Intake, Body Mass Index, and Physical Activity Among Japanese Adults: A Longitudinal Study

**DOI:** 10.3389/fnut.2022.753127

**Published:** 2022-02-08

**Authors:** Daiki Watanabe, Haruka Murakami, Yuko Gando, Ryoko Kawakami, Kumpei Tanisawa, Harumi Ohno, Kana Konishi, Azusa Sasaki, Akie Morishita, Nobuyuki Miyatake, Motohiko Miyachi

**Affiliations:** ^1^Department of Physical Activity Research, National Institute of Health and Nutrition, National Institutes of Biomedical Innovation, Health and Nutrition, Shinjuku-ku, Japan; ^2^Institute for Active Health, Kyoto University of Advanced Science, Kameoka, Japan; ^3^Faculty of Sport and Health Science, Ritsumeikan University, Kusatsu, Japan; ^4^Faculty of Sport Science, Surugadai University, Hanno, Japan; ^5^Faculty of Sport Sciences, Waseda University, Tokorozawa, Japan; ^6^Department of Nutrition, Faculty of Medical and Health Care, Kiryu University, Midori, Japan; ^7^Faculty of Food and Nutritional Sciences, Toyo University, Ora-gun, Japan; ^8^Department of Food and Nutrition, Jumonji University, Niiza, Japan; ^9^Okayama Southern Institute of Health, Okayama Health Foundation, Okayama, Japan; ^10^Faculty of Medicine, Kagawa University, Kita-gun, Japan

**Keywords:** diet quality, longitudinal trajectory, Nutrient-Rich Food Index 9.3, dietary assessment questionnaire, accelerometer, interindividual heterogeneity, lifestyle factors

## Abstract

**Background:**

Many cross-sectional studies have identified modifiable factors such as dietary intake, physique, and physical activity associated with diet quality but were unable to determine how a specific individual's diet quality changes with these factors. These relationships may vary depending on an individual's dietary intake. We aimed to determine the association between temporal changes in diet quality and concurrent changes in dietary intake, body mass index (BMI), and physical activity according to the diet quality trajectory pattern.

**Methods:**

This longitudinal prospective study included 697 Japanese adults aged 26–85 years, at baseline, with available data from at least two dietary intake surveys (4,118 measurements). Dietary intake and physical activity were evaluated using validated dietary questionnaires and a triaxial accelerometer. Diet quality was calculated using the Nutrient-Rich Food Index 9.3 (NRF9.3), while physical activity was calculated based on the duration of activity performed at each level of intensity (sedentary, light, moderate, and vigorous). Body mass index was calculated from the measured height and weight. Statistical analyses involved latent class growth models (LCGM) and random-effect panel data analysis.

**Results:**

During a mean follow-up period of 6.8 years, NRF9.3 scores were assessed, on average, 5.4 times in men and 6.1 times in women. Based on the NRF9.3 score, three separate trajectory groups—“low-increasing,” “medium-increasing,” and “high-stable”—among individuals aged 26–90 years were identified using LCGM. In the multivariate analysis, the NRF9.3 score trajectory was positively associated with intake of energy, protein, dietary fiber, vitamins A and C, magnesium, and food items, such as fruits and vegetables, and was negatively associated with BMI and the intake of added sugar, saturated fats, sodium, and food items, such as meat and sugar and confectioneries, even after adjusting for covariates. These relationships displayed heterogeneity across the identified NRF9.3 score trajectory groups. In the low-increasing group, an inverse relationship was observed between sedentary behavior and NRF9.3 score trajectory.

**Conclusions:**

We identified modifiable factors associated with temporal changes in diet quality across a wide age range; however, these factors may vary according to the diet quality trajectories. Our findings may help develop effective strategies for improving diet quality, according to the trajectory of diet quality.

## Introduction

Worldwide, poor diet quality is a leading and modifiable cause of adverse health outcomes, including non-communicable diseases and maternal and child illnesses (1–3). Comparing diet quality among 187 countries between 1990 and 2010 showed that the consumption of healthy food items improved globally, whereas consumption of unhealthy food items worsened, with heterogeneity across regions and countries (4). The difference in diet quality between countries may explain the large global differences in the non-communicable disease burden (2). Therefore, it is necessary to establish effective public health programs to inculcate appropriate lifestyle behaviors, such as improving the diet quality to prolong the healthy lifespan.

The Nutrient-Rich Food Index 9.3 (NRF9.3) is a nutrient profiling method based on nutrient density rather than food intake (5, 6). The NRF9.3 is a useful, comprehensive system for nutritional guidance and education because the score algorithms can be widely applied to the total diet, specific meals, and individual foods (7). Diets with higher NRF9.3 scores are associated with higher consumption of those foods and nutrients that should be encouraged and a lower total energy intake (8). Epidemiological research has shown that the NRF9.3 score is inversely associated with the risk of overall mortality in adults (9); therefore, it may help consumers identify foods that provide optimal nutrition.

Although diet quality is associated with demographic, socioeconomic, and lifestyle-related factors (10), modifiable risk factors, such as dietary intake, physique, and physical activity, that consider the feasibility of improving the diet quality should be evaluated. Many cross-sectional studies have shown that diet quality is associated with dietary intake (1, 11), body mass index (BMI) (12, 13), and physical activity (13, 14). However, due to their cross-sectional design, these studies are limited to describing population-level changes and not determining how a specific individual's diet quality changes with the associated factors (15). Despite these relationships having been verified by longitudinal studies (10, 16), the associations of changes in diet quality with dietary intake, BMI, and physical activity over time need to be evaluated. Thus far, the effect of dietary intake, BMI, and physical activity on the NRF9.3 score trajectory in adults has not been demonstrated, which is essential for maintaining and improving the diet quality considering the increasingly global spread of the Western lifestyle and obesity (4, 17). In this study, we aimed to (1) evaluate the patterns of longitudinal changes in the NRF9.3 score over time according to age and sex and (2) determine the association between changes in the NRF9.3 score over time and concurrent changes in dietary intake, BMI, and physical activity parameters across a wide age range, for each identified diet quality trajectory pattern, among Japanese adults. We hypothesized that dietary intake, physical activity, and BMI—factors that affect changes in diet quality—may vary depending on participant's dietary characteristics. The results of this study will provide knowledge regarding factors that are likely to provide greater benefits to improving diet quality according to the trajectory of diet quality.

## Materials and Methods

### Study Population

This longitudinal prospective study utilized data from the cohort study, which has been described previously (18, 19). This cohort study has been managed by the National Institute of Health and Nutrition since 2007. It aimed to evaluate the association between lifestyle-related diseases and modifiable risk factors, such as dietary intake and physical activity. In total, 1,075 individuals who participated in a specific health examination conducted at the Okayama Southern Institute of Health or the National Institute of Health and Nutrition were recruited from the Tokyo metropolitan area (*n* = 819) and Okayama prefectures (*n* = 256) between 2007 and 2015. A total of 315 participants were excluded from this study because they met at least one of the following exclusion criteria: history of stroke, cardiac disease, chronic renal failure, or difficulty with ambulation due to knee or back pain and non-renewal of informed consent in 2019. This cohort study included the remaining 760 adults (response rate: 70.7%) aged 26–85 years at baseline, living in the Tokyo metropolitan area (*n* = 504) and Okayama Prefecture (*n* = 256) in Japan.

All participants were requested, by postal mail, to participate in an annual health check-up examination conducted face-to-face and using mail survey. The annual examinations were conducted using the same survey content and methodology, and participants were followed up for a maximum of 12 years from 2007 to 2018. Details regarding the number of participants that were followed up are shown in Supplementary Table 1. This study protocol was approved by the ethics review board of the Research Ethical Review Committee of the National Institute of Health and Nutrition (approval no. kenei102-01). Written informed consent was obtained from all participants before data acquisition.

Of the participants initially included at baseline (*n* = 760), we excluded individuals with missing data on age and sex (*n* = 1), those with outlier values for estimated energy intake [<600 or >4,000 kcal/day (7 and 10 measurements, respectively)] (19), and those who only completed one dietary survey (*n* = 62). The final dataset for analysis included 697 participants (4,118 measurements) for whom dietary information from at least two face-to-face surveys was available (response rate: 64.8%).

### Dietary Assessment

Dietary intake was evaluated using the brief-type self-administered diet history questionnaire (BDHQ), which consists of 58 food and beverage items that have been validated against dietary records (20–22) and comprises items that are commonly consumed, according to the National Health and Nutrition Survey in Japan (23). The BDHQ examined only the frequency of consumption of the food and beverage items in the preceding month. Fixed portion sizes by sex were derived mainly from several recipe books on Japanese cuisine. Participants with unclear responses or unanswered questions confirmed their responses in a face-to-face interview conducted by well-trained registered dietitians. Nutrient intake was calculated from the weight of the items (according to the portion size and frequency of intake) and the nutritional value of the foods and beverages listed in the Standard Tables of Food Composition in Japan (24).

### Evaluation of Physical Activity

As an objective index of physical activity, we used a triaxial accelerometer (EW4800, Panasonic Co., Ltd., Osaka, Japan) to measure the duration of activity undertaken at each intensity level (sedentary, light, moderate, and vigorous). This triaxial accelerometer was previously validated in adults against total energy expenditure measured using the metabolic chamber and the doubly-labeled water methods (25). The research staff was trained to administer the accelerometer to participants using written instructions for its use. Participants were instructed to wear the triaxial accelerometer on their waist for 28 days from when they woke up until bedtime, except while swimming, sleeping, and bathing. The duration of activity at each intensity level per minute, basal metabolic rate, step count, and physical activity level were determined using the manufacturer's algorithm. Data were excluded for low-adherence days when the wearing time, calculated as 24 h minus the non-wearing and no signal time, was <6 h per day. These criteria were confirmed and matched non-wearing time with more than 10 h per day (26) in subgroups. The sum of all physical activities undertaken over at least 7 days (including weekdays and weekend days) was divided by the total number of survey days to obtain the mean daily duration of physical activity. The daily duration of physical activity was categorized as follows: <1.5 metabolic equivalents (METs; sedentary behavior), 1.5–2.9 METs (light intensity), 3.0–5.9 METs (moderate intensity), and ≥6.0 METs (vigorous intensity) (27, 28).

### Calculation of the NRF9.3 Score

The overall diet quality was evaluated using the NRF9.3, as described previously (5, 6, 11). Briefly, the NRF9.3 score consists of values for nine nutrients (protein, total dietary fiber, vitamins A, C, and D, calcium, iron, potassium, and magnesium) to be encouraged (NR9) and three nutrients (added sugars, saturated fats, and sodium) to be limited (LIM3). The NRF9.3 score is calculated as the sum of the percentages of reference daily values (RDVs) for NR9 minus the sum of the percentages of RDVs for LIM3, with the score for each nutrient falling within a range of 0–100. Reference daily values have been determined for sex and age categories based on the Dietary Reference Intakes (DRIs) for Japanese, 2015 (11). These RDVs were adjusted for sex- and age-specific estimated energy requirements based on moderate-level physical activity from the DRIs. The reference values for daily intake according to the DRIs for Japanese 2015, which were to calculate NRF9.3 are presented in Supplementary Table 2. The NR9 and NRF9.3 scores ranged from 0 (poor) to 900 (better), whereas the LIM3 score ranged from 0 (better) to 300 (poor). All nutrient values were derived exclusively from foods and beverages and not from supplements because of the defined intention to assess the nutrient intake exclusively from foods and beverages (11). The Pearson's correlation coefficient between the NRF9.3 score derived from the BDHQ and dietary records was 0.37 in men and 0.61 in women (11). Moreover, the interclass correlation coefficient (ICC) as a measure of the reproducibility of NRF9.3 score estimated by BDHQ was 0.56 for men and 0.77 for women (11).

### Other Covariates

Health information, such as medical history and smoking status, was obtained using self-report structured questionnaires. Research staff reviewed all questionnaires and interviewed respondents with unanswered questions or unclear responses, occasionally confirming answers. Comorbidity scores were calculated from the data obtained on comorbidity status (including hypertension, dyslipidemia, diabetes, ischemic heart disease, other heart diseases, cerebrovascular diseases, renal failure, cancer, osteoporosis, and depression). The summed value indicated a total score ranging from 0 (no comorbidity) to 10 (significant comorbidity). Each participant's body weight was measured while wearing light clothing (Inner Scan BC-600, Tanita Co., Tokyo, Japan). Body mass index was calculated by dividing the measured body weight by the square of the height (kg/m^2^).

### Statistical Analysis

We used latent growth curve models (LGCMs) and latent class growth models (LCGM) to identify longitudinal trajectories from repeated measures of diet quality using the STATA macro TRAJ (29). The LGCMs were used to estimate a single mean diet quality score trajectory across the group in a sex-stratified model to evaluate whether the participants could be classified into multiple trajectory groups using the maximum likelihood method. These models constructed a trajectory shape with a cubic specification. To determine the membership of the trajectory group for each participant, the best-fitting model for the LCGM was identified by estimating models with 2–8 latent clusters and subsequently comparing them by using the sample size of the clusters (≥5%) and the Bayesian Information Criterion as the primary fit index (30).

The NRF9.3 score trajectory was categorized into three groups: low-increasing, *n* = 94; medium-increasing, *n* = 338; and high-stable, *n* = 265 (details are shown in the results). Categorical variables are expressed as numbers and percentages, and the groups were compared using the chi-square test. Continuous variables are expressed as means and standard deviations, and the groups were compared using the analysis of variance. To avoid causing a reduction in efficiency due to the reduced available sample size (selection bias), we performed imputation of missing values for covariates from five data sets created using the multivariate imputation by chained equations using the R software (31): comorbidity score (189 measurements) and physical activity (251 measurements). These missing values were assumed to be missing at random. It has been suggested that 5% missing data is the upper threshold for which multiple imputations provide a benefit in large data sets; if the missing values exceed 10%, it is stated that bias is likely in the analyses (32).

We evaluated the accuracy and precision of the group's mean NRF9.3 score trajectory estimated from the dietary questionnaire using previously reported equations (33). These equations were used to estimate the required sample size and measurement time from within-person variance, between-person variance, and the ratio of within-person to between-person variance (details are provided in the footnote to **Table 2**) that were performed with the sex-stratified cohort data (33).

To evaluate the factors associated with the NRF9.3 score trajectory, we used the random-effect panel data multivariate regression analysis, which evaluates the related factors from the longitudinal changes of the dependent and explanatory variables by adjusting the between-individual characteristics (34). To avoid multicollinearity and evaluate factors associated with the NRF9.3 score trajectory, we investigated the effect size derived from (1) the constituent nutrients in the NRF9.3 and (2) food intake and physical activity. In the abovementioned multivariate models, we have added age (continuous), sex (female or male), region (urban or local), BMI (continuous), comorbidity scores (continuous), smoking status (never, past, or current smoker), and energy intake (continuous). These analyses were also conducted using the generalized estimating equations (GEE) to verify the population-average effects and compare the analysis results based on the same individual and population. These variables were selected with reference to covariates used in previous studies (10, 16). These analyses are shown as regression coefficients with 95% confidence intervals (CIs), calculated for each variable per unit increment. Furthermore, these analyses were conducted after stratification according to the NRF9.3 score trajectory groups. The sensitivity analysis for the results was conducted as a similar analysis using the complete dataset. Moreover, we similarly examined the relationship between the NRF9.3 domains (NR9 and LIM3) and factors.

A two-sided *p*-value < 0.05 was considered significant. All analyses were performed using STATA MP, version 15.0 (StataCorp LP, College Station, TX, USA) and/or R software 3.4.3 (R Core Team, Vienna, Austria).

## Results

### Identifying the NRF9.3 Trajectory Groups

**Figure 1:**
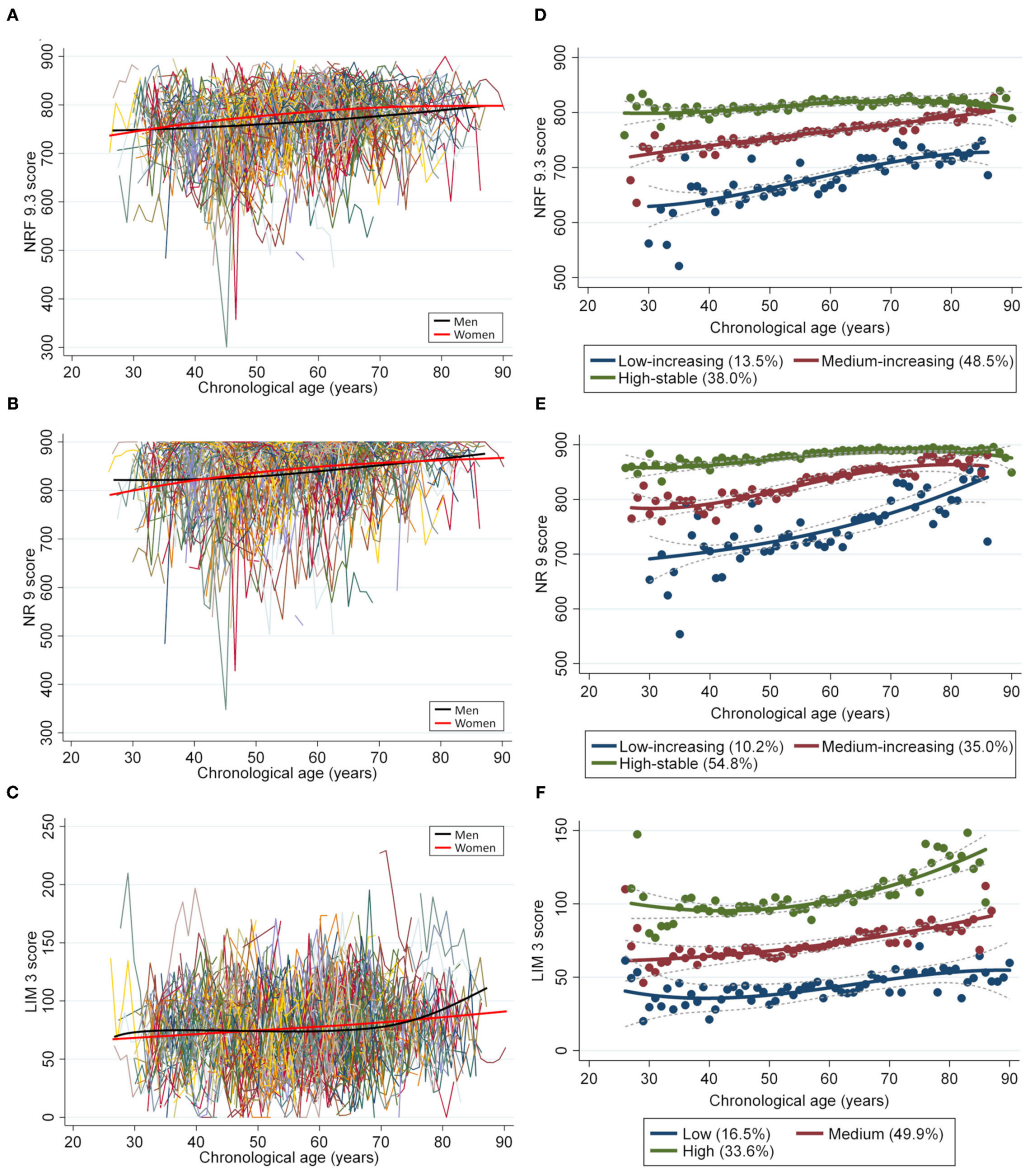
Longitudinal trajectories of the diet quality score in 697 individuals (4,118 measurements). A total of 4,118 repeated measurements from 697 individuals were included to estimate the longitudinal trajectories of diet quality. The latent growth curve models were used to estimate a single mean **(A)** NRF9.3, **(B)** NR9, and **(C)** LIM3 score trajectory across the groups by using a sex-stratified model. Diet quality measurements are presented such that an individual is connected to the same color lines when more than one measurement is assessed for a given individual. The average change in diet quality score with age is indicated by smooth lines (black for men and pink for women). The latent class growth models identified three distinct trajectory groups according to the **(D)** NRF9.3, **(E)** NR9, and **(F)** LIM3 scores in participants aged 26–90 years, using the maximum likelihood method.

During a mean follow-up period of 6.8 years, the NRF9.3 scores were assessed, on average, 5.4 times in men and 6.1 times in women. The single mean NRF9.3 scores trajectory according to chronological age in men and women are presented in Figure 1. With increasing age, the average NRF9.3, NR9, and LIM3 scores slightly increased in both sexes. Moreover, three separate trajectories for the NRF9.3, NR9, and LIM3 scores of the participants, aged 26–90 years, were identified. These trajectories were maintained even after stratification by sex (see Supplementary Figure 1) and were defined as “low-increasing,” “medium-increasing,” and “high-stable” groups according to the NRF9.3 score.

### Participant Characteristics According to the NRF9.3 Trajectory Groups

The low-increasing group (*n* = 94; 13.5%) was characterized by a low NRF9.3 score during young adulthood; the medium-increasing group (*n* = 338; 48.5%), by a moderate NRF9.3 score; and the high-stable group (*n* = 265; 38.0%), by the maintenance of a high NRF9.3 score. The difference in the NRF9.3 scores between the trajectory groups was large in the younger population, although it tended to be smaller with increasing age. Table 1 shows the participant characteristics by the NRF9.3 score trajectory group. Participants in the high-stable group were characterized as more likely to be female, non-smokers, and those with higher physical activity levels; they also had a low LIM3 score. Baseline food and nutrient intakes were significantly different between the NRF9.3 score trajectory groups (see Supplementary Table 3. The included study participants were older than those who were excluded and were more likely to be female; nevertheless, serious bias was not observed (see Supplementary Table 4).

**Table 1:** Baseline demographic and physical activity-related characteristics according to the NRF9.3 trajectory group.

	**Total**		**NRF9.3 score trajectory group**						
			**Low-increasing**		**Medium-increasing**		**High-stable**		***p*-values**
*N* (%)^a^	697	(100)	94	(13.5)	338	(48.5)	265	(38.0)	
Age (years)^b^	52.5	(11.6)	53.3	(11.7)	52.5	(11.6)	52.3	(11.5)	0.747
Women [*n* (%)]^a^	486	(69.7)	52	(55.3)	226	(66.9)	208	(78.5)	<0.001
Local area [*n* (%)]^a^	232	(33.3)	38	(40.4)	103	(30.5)	91	(34.3)	0.174
Body mass index (kg/m^2^)^b^	22.5	(2.9)	22.8	(3.4)	22.5	(2.7)	22.2	(2.9)	0.151
No comorbidity [*n* (%)]^a^	530	(76.0)	65	(69.1)	256	(75.7)	209	(78.9)	0.185
Smoker [*n* (%)]^a^	197	(28.3)	39	(41.5)	104	(30.8)	54	(20.4)	<0.001
Alcohol drinker [*n* (%)]^a^	514	(73.7)	67	(71.3)	260	(76.9)	187	(70.6)	0.179
Energy intake (kcal/day)^b^	1,817	(489)	1,848	(523)	1,807	(507)	1,818	(453)	0.771
Step count (steps/day)^b^	10,192	(3,543)	10,251	(4,161)	10,066	(3,492)	10,331	(3,375)	0.650
BMR (kcal/day)^b^	1,224	(143)	1,275	(140)	1,228	(144)	1,201	(140)	<0.001
TEE (kcal/day)^b^	1,927	(250)	1,976	(256)	1,923	(232)	1,916	(268)	0.124
Physical activity level^b^	1.58	(0.14)	1.55	(0.16)	1.58	(0.13)	1.60	(0.13)	0.009
Sedentary behavior (min/day)^b^	220	(47)	215	(46)	221	(49)	220	(44)	0.613
LPA (min/day)^b^	354	(94)	333	(98)	350	(94)	366	(92)	0.008
MPA (min/day)^b^	57	(25)	55	(30)	56	(24)	59	(24)	0.188
VPA (min/day)^b^	2	(7)	2	(6)	2	(6)	2	(8)	0.808
NR9 score^b^	838	(71)	741	(87)	833	(60)	879	(31)	<0.001
LIM3 score^b^	73	(33)	77	(42)	79	(32)	64	(29)	<0.001
NRF9.3 score^b^	765	(68)	663	(68)	754	(49)	816	(36)	<0.001

*NRF9.3, Nutrient-Rich Food Index 9.3; BMR, basal metabolic rate; TEE, total energy expenditure; LPA, low intensity physical activity; MPA, moderate intensity physical activity; VPA, vigorous intensity physical activity; NR9, nine nutrients to be encouraged (protein, total dietary fiber, vitamins A, C, and D, calcium, iron, potassium, and magnesium); LIM3, three nutrients to be limited (added sugars, saturated fats, and sodium).*

a *Categorical variables are expressed as number and percentage, and groups were compared using the chi-square test.*

b *Continuous variables are expressed as mean and standard deviation and groups were compared using the analysis of variance*.

### Accuracy and Precision of the NRF9.3 Trajectory

Table 2 shows the accuracy and precision of the study group's mean NRF9.3 score trajectory estimated based on data obtained from the dietary questionnaire. Women tended to have a higher NRF9.3 score than men at baseline. The larger values of within-person variance, between-person variance, and the ratio of within-person to between-person variance imply that a more measurement times and larger population are required for an NRF9.3 score trajectory assessment. However, no great sex-based difference was observed in these variances. The interindividual variance in the NRF9.3 score trajectory was 64%. Group sizes required to estimate a group's “true” mean NRF9.3 score trajectory within a 95% CI with a 1% deviation ranged from 291 (women) to 403 (men). Five-time measures of BDHQ were required to obtain a correlation coefficient (*r*) of 0.95 between an individual's evaluated value and his/her “true” unevaluated usual mean NRF9.3 score trajectory.

**Table 2:** Accuracy and precision of the NRF9.3 score according to sex.

	**Total (*n* = 697)** **(4,118 measurements)**	**Men (*n* = 211)** **(1,142 measurements)**	**Women (*n* = 486)** **(2,976 measurements)**
Mean NRF9.3 score (SD) at baseline	765 (68)	743 (71)	775 (65)
CV_w_ (%)^a^	4.5	5.1	4.3
CV_b_ (%)^a^	8.1	8.9	7.6
VR	0.56	0.57	0.57
ICC^b^	0.64	0.64	0.64
Required group size^c^			
Specified % deviation			
1	331	403	291
2.5	53	64	47
5	13	16	12
10	3	4	3
Required measurement times^d^			
Specified correlation coefficient			
0.80	1	1	1
0.85	1	1	1
0.90	2	2	2
0.95	5	5	5
Required measurement times^e^			
Specified % deviation			
1	79	98	72
2.5	13	16	11
5	3	4	3
10	1	1	1

*NRF9.3, Nutrient-Rich Food Index 9.3; SD, standard deviation; CV_w_, coefficient of within-person variation; CV_b_, coefficient of between-person variation; VR, within-person to between-person variance ratio; ICC, intraclass correlation coefficient.*

a *The CV_w_ and CV_b_ for the NRF9.3 score were calculated using analysis of variance.*

b *ICC = CV_b_/(CV_w_ + CV_b_). If the ICC is comparatively high, it means a larger CV_b_ in the NRF9.3 score trajectory.*

c *The group size = 1.96^2^ × [(${CV}_{b}^{2}$ + ${CV}_{w}^{2}$)/$D_{0}^{2}$] required to estimate a group's “true” mean NRF9.3 score trajectory within a 95% confidence interval with a specified % deviation (D_0_). All values indicate group sizes.*

d *The number of measurements during the study period (NT_1_) = [r^2^/(1 – r^2^)] × VR required to obtain a specified r between an individual's measured value and unmeasured usual “true” mean NRF9.3 score trajectory, where r is the specified correlation coefficient and an index of confidence related to an individual's classification or ranking within a population. All values indicate the number of measurements during the study period.*

e *The number of measurements during the study period (NT_2_) = (1.96 × CV_w_/D_1_)^2^ required to estimate an individual's “true” mean NRF9.3 score trajectory within a 95% confidence interval with a specified % deviation (D_1_). All values indicate the number of measurements during the study period*.

### Factors Associated With the NRF9.3 Trajectories by Multivariate Analysis

We evaluated the relationship between the NRF9.3 score trajectory and constituent nutrients in the NRF9.3 using multivariate analysis (Table 3). In the analysis of the entire study cohort, the NRF9.3 score trajectory was positively associated with the intake of energy, protein, dietary fiber, vitamins A and C, and magnesium, and negatively associated with BMI and the intake of added sugar, saturated fats, and sodium. Although associations were found between sex and the intake of dietary fiber and sodium in all the NRF9.3 score trajectory groups, some nutrients indicated heterogeneity across the identified NRF9.3 score trajectory groups. The increased intake of saturated fatty acids by participants had greater adverse effects on the NRF9.3 score in the high-stable group than in the low-increasing group. However, the beneficial effects of dietary fiber and vitamin A and C intake were reversed. Associations of the NRF9.3 score change with dietary intake and physical activity are shown in Table 4. We demonstrated that the NRF9.3 score trajectory is positively associated with the consumption of pulses, potatoes, vegetables (other than pickled vegetables), mushrooms, fruits, eggs, dairy products, coffee, and fruit and vegetable juice and negatively associated with the consumption of pickled vegetables, meat, soft drinks, and sugar and confectioneries, even after adjusting for covariates. However, these relationships indicated heterogeneity across the identified NRF9.3 score trajectory groups, similar to the nutrient results shown in Table 3. In the low-increasing group, we found an inverse relationship between sedentary behavior and the NRF9.3 score trajectory. Similar results were obtained for the complete case analysis, after excluding data with missing values, in the sensitivity analysis (see Supplementary Table 5). Sedentary behavior was associated with a low NR9 score trajectory in the low-increasing group but not with the LIM3 score (see Supplementary Tables 6, 7). In addition, the results for panel data analysis (change in variables in the same individuals over time) and GEE (population-averaged effects) were different (see Supplementary Tables 8, 9).

**Table 3:** Associations between change in the NRF9.3 score and constituent nutrients using multivariate panel data analysis.

**Increment effects/unit**	**Total**		**NRF9.3 score trajectory group**					
			**Low-increasing**		**Medium-increasing**		**High-stable**	
	**RC**	**95% CI**	**RC**	**95% CI**	**RC**	**95% CI**	**RC**	**95% CI**
*N* (measurement)	697 (4,118)		94 (531)		338 (2,007)		265 (1,580)	
Within *R*^2^	*R^2^* = 0.392		*R^2^* = 0.740		*R^2^* = 0.488		*R^2^* = 0.535	
Age (1 year)	0.236	(0.032 to 0.439)^*^	−0.342	(−0.974 to 0.290)	0.449	(0.272 to 0.624)^*^	0.487	(0.351 to 0.623)^*^
Women	−7.611	(−13.542 to −1.679)^*^	−36.457	(−52.034 to −20.879)^*^	−11.897	(−16.628 to −7.165)^*^	5.383	(1.158 to 9.606)^*^
Local area	−0.667	(−5.913 to 4.579)	1.889	(−13.000 to 16.779)	2.699	(−1.578 to 6.976)	1.145	(−2.015 to 4.305)
Body mass index (1 kg/m^2^)	−0.760	(−1.481 to −0.038)^*^	−0.777	(−2.631 to 1.078)	−0.096	(−0.733 to 0.541)	−0.272	(−0.726 to 0.181)
Comorbidity score (1 point)	−0.920	(−2.892 to 1.051)	4.351	(−1.535 to 10.236)	−0.534	(−2.683 to 1.615)	−1.640	(−3.303 to 0.024)
Smoker	−3.792	(−8.263 to 0.679)	−4.018	(−16.492 to 8.456)	−0.049	(−4.075 to 3.978)	−2.322	(−5.891 to 1.247)
Energy intake (1 kcal)	0.004	(0.000 to 0.007)^*^	0.004	(−0.005 to 0.012)	0.007	(0.003 to 0.010)^*^	0.002	(0.000 to 0.005)^*^
Protein (1 % energy)	2.916	(1.689 to 4.141)^*^	1.954	(−1.675 to 5.583)	1.280	(−0.125 to 2.685)	−0.125	(−1.272 to 1.022)
Dietary fiber (1 g/1,000 kcal)	8.126	(6.641 to 9.610)^*^	20.232	(15.113 to 25.351)^*^	7.627	(5.875 to 9.377)^*^	4.426	(3.103 to 5.747)^*^
Vitamin A (1 μg RAE/1,000 kcal)	0.020	(0.012 to 0.026)^*^	0.040	(0.021 to 0.059)^*^	0.026	(0.017 to 0.034)^*^	0.005	(−0.001 to 0.012)
Vitamin C (1 mg/1,000 kcal)	0.242	(0.140 to 0.342)^*^	0.623	(0.259 to 0.986)^*^	0.204	(0.076 to 0.331)^*^	0.033	(−0.046 to 0.112)
Vitamin D (1 μg/1,000 kcal)	0.009	(−0.524 to 0.542)	0.968	(−0.630 to 2.566)	0.092	(−0.530 to 0.714)	0.100	(−0.400 to 0.600)
Calcium (1 mg/1,000 kcal)	−0.004	(−0.029 to 0.020)	0.036	(−0.048 to 0.120)	−0.010	(−0.038 to 0.018)	0.034	(0.011 to 0.056)^*^
Iron (1 mg/1,000 kcal)	2.220	(−0.958 to 5.399)	8.719	(−3.409 to 20.846)	1.664	(−2.157 to 5.485)	4.619	(2.013 to 7.224)^*^
Potassium (1 mg/1,000 kcal)	0.012	(−0.002 to 0.027)	0.089	(0.037 to 0.140)^*^	0.017	(−0.001 to 0.034)	0.004	(−0.008 to 0.016)
Magnesium (1 mg/1,000 kcal)	0.352	(0.171 to 0.533)^*^	0.076	(−0.492 to 0.643)	0.366	(0.163 to 0.568)^*^	0.020	(−0.139 to 0.180)
Added sugars (1 % energy)	−1.927	(−2.836 to −1.017)^*^	−5.863	(−8.054 to −3.671)^*^	−0.464	(−1.472 to 0.545)	−2.693	(−3.565 to −1.820)^*^
Saturated fats (1 % energy)	−3.985	(−5.061 to −2.907)^*^	−1.730	(−4.862 to 1.403)	−4.167	(−5.364 to −2.968)^*^	−5.244	(−6.313 to −4.175)^*^
Sodium (1 mg/1,000 kcal)	−0.048	(−0.051 to −0.043)^*^	−0.044	(−0.056 to −0.032)^*^	−0.036	(−0.040 to −0.030)^*^	−0.054	(−0.057 to −0.049)^*^

*NRF9.3, Nutrient-Rich Food Index 9.3; RC, regression coefficient; CI, confidence interval. The results of these analyses are presented as RC and 95% CI, which were calculated for each variable per unit increment.*

The asterisk (*) indicates statistical significance (p < 0.05). Sex and area were time-stable variables, while the other covariates were time-varying variables.

**Table 4:** Associations of change in the NRF9.3 score with dietary intake and physical activity using multivariate panel data analysis.

**Increment effects/unit**	**Total**		**NRF9.3 score trajectory group**					
			**Low-increasing**		**Medium-increasing**		**High-stable**	
	**RC**	**95% CI**	**RC**	**95% CI**	**RC**	**95% CI**	**RC**	**95% CI**
*N* (measurement)	697 (4,118)		94 (531)		338 (2,007)		265 (1,580)	
Within *R*^2^	*R^2^* = 0.319		*R^2^* = 0.694		*R^2^* = 0.400		*R^2^* = 0.364	
Age (1 year)	0.054	(−0.164 to 0.273)	−0.301	(−0.956 to 0.353)	0.309	(0.111 to 0.506)^*^	0.308	(0.137 to 0.479)^*^
Women	1.006	(−5.544 to 7.556)	−28.199	(−44.97 to −11.427)^*^	−7.127	(−12.65 to −1.604)^*^	12.268	(6.932 to 17.603)^*^
Local area	−0.758	(−6.391 to 4.875)	−14.313	(−29.807 to 1.181)	2.607	(−2.143 to 7.357)	3.130	(−0.770 to 7.030)
Body mass index (1 kg/m^2^)	−1.129	(−1.896 to −0.360)^*^	−0.012	(−1.906 to 1.883)	−0.174	(−0.859 to 0.512)	−0.743	(−1.301 to −0.184)^*^
Comorbidity score (1 point)	−0.678	(−2.780 to 1.425)	−0.958	(−7.442 to 5.525)	−0.527	(−2.849 to 1.795)	−0.810	(−2.837 to 1.216)
Smoker	−4.508	(−9.268 to 0.252)	−0.672	(−13.607 to 12.263)	−2.573	(−6.894 to 1.749)	0.396	(−3.943 to 4.735)
Energy intake (1 kcal)	0.017	(0.012 to 0.020)^*^	0.014	(0.004 to 0.023)^*^	0.014	(0.010 to 0.018)^*^	0.014	(0.009 to 0.017)^*^
Cereals (1 g/1,000 kcal)	0.056	(−0.001 to 0.113)	0.134	(−0.009 to 0.276)	0.029	(−0.034 to 0.092)	0.038	(−0.028 to 0.104)
Pulses (1 g/1,000 kcal)	0.442	(0.371 to 0.513)^*^	1.071	(0.831 to 1.311)^*^	0.349	(0.265 to 0.431)^*^	0.179	(0.109 to 0.248)^*^
Potatoes (1 g/1,000 kcal)	0.499	(0.430 to 0.568)^*^	1.041	(0.743 to 1.338)^*^	0.435	(0.345 to 0.524)^*^	0.253	(0.188 to 0.317)^*^
Green and yellow vegetables (1 g/1,000 kcal)	0.423	(0.377 to 0.468)^*^	1.196	(0.994 to 1.398)^*^	0.370	(0.313 to 0.427)^*^	0.219	(0.176 to 0.262)^*^
Other vegetables (1 g/1,000 kcal)	0.193	(0.153 to 0.232)^*^	0.758	(0.598 to 0.918)^*^	0.189	(0.135 to 0.242)^*^	0.100	(0.064 to 0.135)^*^
Pickled vegetables (1 g/1,000 kcal)	−0.210	(−0.369 to −0.050)^*^	0.504	(−0.007 to 1.016)	−0.067	(−0.260 to 0.127)	−0.593	(−0.750 to −0.435)^*^
Mushrooms (1 g/1,000 kcal)	0.432	(0.152 to 0.711)^*^	−0.495	(−1.641 to 0.651)	0.092	(−0.254 to 0.438)	0.176	(−0.076 to 0.428)
Seaweeds (1 g/1,000 kcal)	−0.110	(−0.332 to 0.112)	−0.372	(−1.102 to 0.358)	0.127	(−0.147 to 0.401)	−0.232	(−0.445 to −0.019)^*^
Fruits (1 g/1,000 kcal)	0.284	(0.241 to 0.325)^*^	0.639	(0.485 to 0.792)^*^	0.257	(0.205 to 0.307)^*^	0.101	(0.061 to 0.141)^*^
Fish and shellfish (1 g/1,000 kcal)	−0.069	(−0.151 to 0.013)	0.437	(0.187 to 0.687)^*^	−0.026	(−0.118 to 0.066)	−0.447	(−0.533 to −0.360)^*^
Meat (1 g/1,000 kcal)	−0.138	(−0.241 to −0.034)^*^	0.217	(−0.066 to 0.500)	−0.196	(−0.313 to −0.078)^*^	−0.381	(−0.496 to −0.264)^*^
Eggs (1 g/1,000 kcal)	0.218	(0.096 to 0.339)^*^	0.662	(0.287 to 1.037)^*^	0.156	(0.020 to 0.292)^*^	−0.042	(−0.166 to 0.082)
Dairy products (1 g/1,000 kcal)	0.048	(0.017 to 0.078)^*^	0.213	(0.119 to 0.306)^*^	0.005	(−0.029 to 0.039)	−0.015	(−0.048 to 0.018)
Oil (1 g/1,000 kcal)	−0.175	(−0.599 to 0.249)	−0.511	(−1.680 to 0.658)	−0.135	(−0.628 to 0.358)	−0.651	(−1.116 to −0.184)^*^
Green tea (1 g/1,000 kcal)	0.039	(0.026 to 0.050)^*^	0.055	(0.015 to 0.093)^*^	0.023	(0.010 to 0.036)^*^	0.013	(0.001 to 0.024)^*^
Black and oolong tea (1 g/1,000 kcal)	0.009	(−0.005 to 0.023)	−0.003	(−0.047 to 0.040)	0.002	(−0.014 to 0.019)	0.002	(−0.011 to 0.016)
Coffee (1 g/1,000 kcal)	0.022	(0.007 to 0.036)^*^	0.077	(0.037 to 0.116)^*^	0.026	(0.011 to 0.041)^*^	0.000	(−0.015 to 0.014)
Fruit and vegetable juice (1 g/1,000 kcal)	0.141	(0.112 to 0.169)^*^	0.313	(0.225 to 0.401)^*^	0.144	(0.108 to 0.180)^*^	0.037	(0.009 to 0.065)^*^
Soft drinks (1 g/1,000 kcal)	−0.035	(−0.067 to −0.003)^*^	0.041	(−0.023 to 0.105)	−0.063	(−0.106 to −0.019)^*^	0.002	(−0.034 to 0.037)
Alcoholic beverages (1 g/1,000 kcal)	−0.001	(−0.025 to 0.022)	0.031	(−0.030 to 0.092)	0.017	(−0.007 to 0.040)	−0.008	(−0.037 to 0.020)
Sugar and confectioneries (1 g/1,000 kcal)	−0.242	(−0.360 to −0.124)^*^	−0.030	(−0.320 to 0.259)	−0.198	(−0.326 to −0.068)^*^	−0.346	(−0.488 to −0.202)^*^
Sedentary behavior (1 min/day)	−0.002	(−0.037 to 0.033)	−0.114	(−0.212 to −0.014)^*^	0.000	(−0.035 to 0.034)	0.029	(−0.006 to 0.063)
LPA (1 min/day)	−0.009	(−0.030 to 0.011)	−0.004	(−0.064 to 0.056)	−0.008	(−0.029 to 0.012)	−0.009	(−0.028 to 0.009)
MPA (1 min/day)	−0.009	(−0.077 to 0.059)	−0.167	(−0.354 to 0.021)	0.009	(−0.066 to 0.084)	−0.006	(−0.068 to 0.056)
VPA (1 min/day)	0.061	(−0.149 to 0.270)	0.719	(−0.051 to 1.490)	−0.283	(−0.564 to 0.001)	0.070	(−0.077 to 0.217)

*NRF9.3, Nutrient-Rich Food Index 9.3; RC, regression coefficient; CI, confidence interval; LPA, low intensity physical activity; MPA, moderate intensity physical activity; VPA, vigorous intensity physical activity. The results of these analyses are presented as RC and 95% CI, which were calculated for each variable per unit increment.*

The asterisk (*) indicates statistical significance (p < 0.05). Sex and area were time-stable variables, while the other covariates were time-varying variables.

## Discussion

In this study, we identified the foods and nutrients that should be encouraged or limited to improve the diet quality by using the NRF9.3 score trajectory in Japanese adults. These relationships indicated heterogeneity across the identified NRF9.3 score trajectory groups. The study's results indicate that an increasing BMI is associated with a decreasing NRF9.3 score. In participants with lower NRF9.3 scores during young adulthood (low-increasing group), increased sedentary behavior was associated with a decreased diet quality. To the best of our knowledge, this is the first study to verify the effect of dietary intake, BMI, and physical activity on the NRF9.3 score trajectory as an indicator of diet quality. This study included participants across a wide age range, and advice on lifestyle changes based on these insights could potentially effectively increase the diet quality of adults.

We identified three separate trajectory groups for diet quality across a wide age range. These groups significantly differed in terms of the proportion of women. However, the same trajectory groups were maintained, even after stratification by sex. The three distinct trajectory groups of diet quality in both sexes demonstrate that it is unlikely for individuals to have similar diet quality trajectories. Based on a comparison of the identified NRF9.3 score trajectory groups, we showed that the mean NRF9.3 score trajectory differed more among younger adults than older adults. Previous comparative dietary intake studies among 187 countries have shown that older adults have better dietary patterns than younger adults (4), and older adults may have smaller interindividual differences than younger adults. However, our finding may be partially attributable to regression toward the mean and “floor and ceiling effects” in the NRF9.3 score (15). Therefore, the increase in the diet quality of a participant with a lower diet quality tends to be exaggerated, and the increase in diet quality of a participant with a higher initial diet quality tends to be dampened. Furthermore, this study included participants who had certain diseases, such as hypertension, diabetes, dyslipidemia, and heart disease, and may have modified their diet to follow each guideline for managing these diseases. However, the results were similar even when the analysis was performed after excluding individuals with these diseases at baseline. In addition, the comorbidity score was not associated with the diet quality score trajectory. Therefore, although it is necessary to re-evaluate these results using a diet quality score other than the NRF9.3 score in a well-designed prospective study, our findings suggest that it is important to provide individuals with opportunities for nutrition education and dietary assessment that could lead to the identification of poor diet quality in younger adults.

We demonstrated associations between changes in the NRF9.3 scores and consumption of dietary fiber, sodium, pulses, potatoes, vegetables, fruits, green tea, and fruit and vegetable juice in all the NRF9.3 score trajectory groups. People from East Asia, including Japan, have a lower intake of fiber, legumes, and fruits and a higher intake of salt than people from other countries (2). These findings may support these dietary intakes results, where both excess and deficiency are problems in the Japanese population associated with changes in diet quality. However, the association between the NRF9.3 score and some dietary intakes indicated heterogeneity across the NRF9.3 score trajectory groups. For example, the increased intake of saturated fatty acids, fish and shellfish, and meat by participants had greater adverse effects on the NRF9.3 score in the high-stable group than in the low-increasing group, although the beneficial effects of dietary fiber, vitamin A and C, pulse, vegetable, and fruit consumption were reversed. A secondary analysis of the PREMIER study showed that improving diet quality by increasing dairy and fruit consumption when following the Dietary Approaches to Stop Hypertension (DASH) diet was more effective in reducing blood pressure and body weight in individuals whose baseline diet was less consistent with the DASH than in those with high adherence to the DASH (35). This indicates that the foods and nutrients that affect these outcomes may vary depending on the individual's dietary intake, and beneficial effects are likely to be greater among individuals at a higher risk for nutrient excesses or deficiencies. Therefore, it is important to evaluate the dietary intake of each individual to determine the applicable educational content (36) because the effectiveness of each nutrient in improving the diet quality differs in each trajectory group.

Our results indicate that an increasing BMI is associated with a decreasing NRF9.3 score in all participants, even after adjusting for energy intake and physical activity. Previous studies have reported that a higher BMI is associated with lower diet quality in cross-sectional (12, 13) and longitudinal (10, 16) studies, and our results were similar. However, while a decrease in diet quality is associated with an increase in BMI, it is impossible to determine whether obesity decreases diet quality or vice versa. In this causal relationship model, the contents to facilitate health guidance may change depending on which comes first. Nevertheless, regardless of which comes first, both the improvement of diet quality (37) and maintaining a healthy BMI (38) are important for preventing the risk of adverse events, such as total and cause-specific mortality.

Several cross-sectional studies have shown that diet quality is positively associated with physical activity (13, 14), although our study did not find this. Our result showed significant differences in physical activity between the diet quality trajectory groups at baseline; these relationships in the cross-sectional studies may have been influenced by interindividual variance. Moreover, a randomized controlled trial has reported increased physical activity in the diet quality intervention group and the control group that did not receive special guidance regarding physical activity (39). This may be because increased physical activity is not directly associated with improved diet quality, as participants may have experienced the effects of increased health consciousness due to study participation. In participants in the low-increasing group, we showed an inverse relationship between sedentary behavior and the NRF9.3 and NR9 score trajectories. Increased sedentary behavior is strongly associated with obesity (40). Although a detailed study of the lifestyles associated with sedentary behavior is needed, it may also be important to replace sedentary behavior with active time; this could be associated with not only lower risk of adverse health outcomes (41) but may also be necessary to improve the diet quality. Education regarding breaks in sedentary time is necessary for some people with poor diet quality in young adulthood because of the association between decreasing diet quality and increasing sedentary behavior.

The strength of this study is the repetitive evaluation of diet quality and physical activity using a validated tool in the same population, which provided an opportunity to examine the association between temporal changes in dietary intake and physical activity parameters and concurrent changes in diet quality across a wide age range. By using the data described above, we could minimize the influence of bias derived from interindividual variance or cohort effects. In addition, we demonstrated that the results of the panel data analysis and GEE varied. These results prove that intra-individual and inter-individual variability of the measured exposure variables may have influenced the analyzed results. However, this study has certain methodological limitations. First, the participants may have been more health-conscious than the general population because we could not select study participants using a random sampling method. In addition, there were differences in the characteristics, such as age and sex, between the included participants and those who were excluded from this study. Therefore, selection bias may have been introduced. Second, we were unable to eliminate measurement bias and confounders completely. Although we examined the medication history and economic status, we could not use these factors as covariates because almost all the data contained missing values. If multiple imputation is performed despite having more than 10% missing values, bias is likely in the analyses (32). Dietary intake estimated using a self-report questionnaire may have been affected by systematic errors due to BMI and recall bias (42). We used a BDHQ that contained only 58 food and beverage items and exclusively assessed intake frequency, not portion size, to evaluate dietary intake. Given that the NRF9.3 score is based on specific absolute cut-offs for dietary components, it is expected to be less accurate in assessing absolute intakes than ranking participants. Although a previous study has reported no significant difference in the mean intake estimated using the four times and single survey from the BDHQ, the reliability of dietary intake estimated from the BDHQ has not been evaluated yet. The accuracy of estimating some nutrient intakes, including energy intakes, is relatively low (20); thus, careful interpretation of our results is necessary. In addition, we did not identify individuals with a decreasing diet quality or low diet quality trajectory pattern; this may be due to the NRF9.3 score, which focuses on nutrient intake and DRIs. Moreover, there may be a potential age-dependent regression to the mean issue that increased the NRF 9.3 scores in the low-increasing trajectory group. It should be noted that we could only calculate the NRF9.3 score using the BDHQ, of which the diet quality score has previously been validated against that calculated from dietary records (11). It is necessary to re-evaluate these results using a diet quality score other than the NRF9.3 score or a more accurate dietary assessment method. Third, the follow-up period was relatively short, and this might have influenced the diet quality trajectory. However, the accuracy and precision of our group's mean NRF9.3 score trajectory were adequate considering the number of conducted surveys (five during the study period) and sample size used in our study. These limitations may prevent the generalization of our results. Therefore, it is necessary to conduct a well-designed prospective cohort study with a larger randomized sample to further assess the effects of dietary intake, BMI, and physical activity on the diet quality trajectory.

In conclusion, we identified foods and nutrients associated with temporal changes in diet quality using the NRF9.3 score trajectory in Japanese adults; however, these relationships indicated heterogeneity across the identified NRF9.3 score trajectory groups. Our findings may provide knowledge to aid the understanding of foods and nutrients to be encouraged or limited to improve the diet quality across a wide age range. Furthermore, our results indicate that an increasing BMI is associated with a decreasing NRF9.3 score. In participants with lower NRF9.3 scores during young adulthood, increased sedentary behavior was associated with a decreased diet quality. Given the heterogeneity of diet quality across regions and countries, there is an urgent need to highlight the importance of selecting optimal foods and behaviors to improve diet quality worldwide. Therefore, these results may provide useful insights into developing effective strategies for improving diet quality according to the trajectory of diet quality.

## Data Availability Statement

The datasets used and analyzed during the current study are available from the corresponding author on reasonable request (cardiovaecular0327@mac.com).

## Ethics Statement

The studies involving human participants were reviewed and approved by the Research Ethical Review Committee of the National Institute of Health and Nutrition. The patients/participants provided their written informed consent to participate in this study.

## Author Contributions

DW and MM formulated the research questions, designed the study, and drafted the manuscript. HM, YG, RK, KT, HO, KK, AS, AM, NM, and MM obtained the data. DW analyzed the data, designed the research, and wrote the paper. HM, YG, RK, KT, HO, KK, AS, AM, and NM provided critical feedback. MM had the primary responsibility for the final contents. All authors read and approved the final manuscript.

## Funding

This study was funded by Health and Labour Sciences Research Grant (200825016B and 201222028B) to MM and Danone Institute of Japan Foundation Young Researchers Grant (DIJF R03-027) to DW for the purpose of designing the study, and for data collection and analysis.

## Conflict of Interest

The authors declare that the research was conducted in the absence of any commercial or financial relationships that could be construed as a potential conflict of interest. The reviewer MY declared a shared affiliation with one of the authors KK at the time of the review.

## Publisher's Note

All claims expressed in this article are solely those of the authors and do not necessarily represent those of their affiliated organizations, or those of the publisher, the editors and the reviewers. Any product that may be evaluated in this article, or claim that may be made by its manufacturer, is not guaranteed or endorsed by the publisher.

## References

[1] Miller V Webb P Micha R Mozaffarian D Global Dietary D. Defining diet quality: a synthesis of dietary quality metrics and their validity for the double burden of malnutrition. Lancet Planet Health. (2020) 4:e352–70. 10.1016/S2542-5196(20)30162-5 32800153 PMC7435701

[2] Collaborators GBDD. Health effects of dietary risks in 195 countries, 1990–2017: a systematic analysis for the Global Burden of Disease Study 2017. Lancet. (2019) 393:1958–72. 10.1016/S0140-6736(19)30041-8 30954305 PMC6899507

[3] Collaborators GBDRF. Global, regional, and national comparative risk assessment of 84 behavioural, environmental and occupational, and metabolic risks or clusters of risks for 195 countries and territories, 1990–2017: a systematic analysis for the Global Burden of Disease Study 2017. Lancet. (2018) 392:1923–94. 10.1016/S0140-6736(18)32225-6 30496105 PMC6227755

[4] Imamura F Micha R Khatibzadeh S Fahimi S Shi P Powles J. Dietary quality among men and women in 187 countries in 1990 and 2010: a systematic assessment. Lancet Glob Health. (2015) 3:e132–42. 10.1016/S2214-109X(14)70381-X 25701991 PMC4342410

[5] Drewnowski A Fulgoni VL III. Nutrient density: principles and evaluation tools. Am J Clin Nutr. (2014) 99:1223S−8S. 10.3945/ajcn.113.073395 24646818

[6] Drewnowski A. The Nutrient Rich Foods Index helps to identify healthy, affordable foods. Am J Clin Nutr. (2010) 91:1095S−101S. 10.3945/ajcn.2010.28450D 20181811

[7] Miller GD Drewnowski A Fulgoni V Heaney RP King J Kennedy E. It is time for a positive approach to dietary guidance using nutrient density as a basic principle. J Nutr. (2009) 139:1198–202. 10.3945/jn.108.100842 19339707

[8] Fulgoni VL III Keast DR Drewnowski A. Development and validation of the nutrient-rich foods index: a tool to measure nutritional quality of foods. J Nutr. (2009) 139:1549–54. 10.3945/jn.108.101360 19549759

[9] Streppel MT Sluik D van Yperen JF Geelen A Hofman A Franco OH. Nutrient-rich foods, cardiovascular diseases and all-cause mortality: the Rotterdam study. Eur J Clin Nutr. (2014) 68:741–7. 10.1038/ejcn.2014.35 24642783

[10] Park SY Shvetsov YB Kang M Setiawan VW Wilkens LR Le Marchand L. Changes in Diet Quality over 10 Years Are Associated with Baseline Sociodemographic and Lifestyle Factors in the Multiethnic Cohort Study. J Nutr. (2020) 150:1880–8. 10.1093/jn/nxaa102 32338763 PMC7330479

[11] Murakami K Livingstone MBE Fujiwara A Sasaki S. Reproducibility and relative validity of the healthy eating index-2015 and nutrient-rich food index 9.3 estimated by comprehensive and brief diet history questionnaires in Japanese adults. Nutrients. (2019) 11:2540. 10.3390/nu11102540 31640242 PMC6836176

[12] Drewnowski A Aggarwal A Tang W Hurvitz PM Scully J Stewart O. Obesity, diet quality, physical activity, and the built environment: the need for behavioral pathways. BMC Public Health. (2016)16:1153. 10.1186/s12889-016-3798-y 27832766 PMC5105275

[13] Pate RR Taverno Ross SE Liese AD Dowda M. Associations among physical activity, diet quality, and weight status in US adults. Med Sci Sports Exerc. (2015) 47:743–50. 10.1249/MSS.0000000000000456 25058328 PMC4422397

[14] XuFCohenSALofgrenIEGreeneGWDelmonicoMJGreaneyML. Relationship between diet quality, physical activity and health-related quality of life in older adults: findings from 2007–2014 national health and nutrition examination survey. J Nutr Health Aging. (2018) 22:1072–9. 10.1007/s12603-018-1050-430379305

[15] Kallman DA Plato CC Tobin JD. The role of muscle loss in the age-related decline of grip strength: cross-sectional and longitudinal perspectives. J Gerontol. (1990) 45:M82–8. 10.1093/geronj/45.3.m82 2335723

[16] Zamora D Gordon-Larsen P Jacobs DR Jr Popkin BM. Diet quality and weight gain among black and white young adults: the Coronary Artery Risk Development in Young Adults (CARDIA) Study (1985–2005). Am J Clin Nutr. (2010) 92:784–93. 10.3945/ajcn.2010.29161 20685947 PMC2937583

[17] Collaboration NCDRF. Rising rural body-mass index is the main driver of the global obesity epidemic in adults. Nature. (2019) 569:260–4. 10.1038/s41586-019-1171-x 31068725 PMC6784868

[18] Gando Y Yamamoto K Murakami H Ohmori Y Kawakami R Sanada K. Longer time spent in light physical activity is associated with reduced arterial stiffness in older adults. Hypertension. (2010) 56:540–6. 10.1161/HYPERTENSIONAHA.110.156331 20606102

[19] Watanabe D Murakami H Ohno H Tanisawa K Konishi K Tsunematsu Y. Association between dietary intake and the prevalence of tumourigenic bacteria in the gut microbiota of middle-aged Japanese adults. Sci Rep. (2020) 10:15221. 10.1038/s41598-020-72245-7 32939005 PMC7495490

[20] Kobayashi S Honda S Murakami K Sasaki S Okubo H Hirota N. Both comprehensive and brief self-administered diet history questionnaires satisfactorily rank nutrient intakes in Japanese adults. J Epidemiol. (2012) 22:151–9. 10.2188/jea.je20110075 22343326 PMC3798594

[21] Kobayashi S Murakami K Sasaki S Okubo H Hirota N Notsu A. Comparison of relative validity of food group intakes estimated by comprehensive and brief-type self-administered diet history questionnaires against 16 d dietary records in Japanese adults. Public Health Nutr. (2011) 14:1200–11. 10.1017/S1368980011000504 21477414

[22] Fujiwara A Murakami K Sasaki S. Relative validity of starch and sugar intake in japanese adults as estimated with comprehensive and brief self-administered diet history questionnaires. J Epidemiol. (2020) 30:315–25. 10.2188/jea.JE20190026 31257352 PMC7348079

[23] Sasaki S Yanagibori R Amano K. Self-administered diet history questionnaire developed for health education: a relative validation of the test-version by comparison with 3-day diet record in women. J Epidemiol. (1998) 8:203–15. 10.2188/jea.8.203 9816812

[24] Council Council for Science and Technology; Ministry of Education Culture Sports Science and Technology Japan. Standard Tables of Food Composition in Japan, Fifth Revised and Enlarged Edition. Tokyo: National Printing Bureau (2005). (In Japanese).

[25] Murakami H Kawakami R Nakae S Nakata Y Ishikawa-Takata K Tanaka S. Accuracy of wearable devices for estimating total energy expenditure: comparison with metabolic chamber and doubly labeled water method. JAMA Intern Med. (2016) 176:702–3. 10.1001/jamainternmed.2016.0152 26999758

[26] Tudor-Locke C Camhi SM Troiano RP. A catalog of rules, variables, and definitions applied to accelerometer data in the National Health and Nutrition Examination Survey, 2003–2006. Prev Chronic Dis. (2012) 9:E113. 10.5888/pcd9.110332 22698174 PMC3457743

[27] Matthews CE Hagstromer M Pober DM Bowles HR. Best practices for using physical activity monitors in population-based research. Med Sci Sports Exerc. (2012) 44:S68–76. 10.1249/MSS.0b013e3182399e5b 22157777 PMC3543867

[28] Chastin SFM De Craemer M De Cocker K Powell L Van Cauwenberg J Dall P. How does light-intensity physical activity associate with adult cardiometabolic health and mortality? Systematic review with meta-analysis of experimental and observational studies. Br J Sports Med. (2019) 53:370–6. 10.1136/bjsports-2017-097563 29695511 PMC6579499

[29] Bobby L. Jones DSN. A note on a stata plugin for estimating group-based trajectory models. Sociol Method Res. (2013) 42:1–6. 10.1177/0049124113503141

[30] Zheng W Mu J Chu C Hu J Yan Y Ma Q. Association of blood pressure trajectories in early life with subclinical renal damage in middle age. J Am Soc Nephrol. (2018) 29:2835–46. 10.1681/ASN.2018030263 30420422 PMC6287870

[31] van Buuren S Groothuis-Oudshoorn K. mice: Multivariate imputation by chained equations in R. J Stat Softw. (2011) 45:1–68. 10.18637/jss.v045.i03

[32] Madley-Dowd P Hughes R Tilling K Heron J. The proportion of missing data should not be used to guide decisions on multiple imputation. J Clin Epidemiol. (2019) 110:63–73. 10.1016/j.jclinepi.2019.02.016 30878639 PMC6547017

[33] Watanabe D Yoshida T Watanabe Y Yamada Y Kimura M Group KS. Objectively measured daily step counts and prevalence of frailty in 3,616 older adults. J Am Geriatr Soc. (2020) 68:2310–8. 10.1111/jgs.16655 33269469 PMC7689814

[34] Baltagi BH Kao C. Nonstationary panels, cointegration in panels and dynamic panels: a survey. In: Baltagi BH, Fomby TB, Carter Hill R. Nonstationary Panels, Panel Cointegration, and Dynamic Panels. Advances in Econometrics, Vol. 15. Bingley: Emerald Group Publishing Limited (2001). p. 7–51.

[35] Pickering RT Bradlee ML Singer MR Moore LL. Baseline diet modifies the effects of dietary change. Br J Nutr. (2020) 123:951–8. 10.1017/S0007114520000112 31959264 PMC7058501

[36] Vadiveloo M Lichtenstein AH Anderson C Aspry K Foraker R Griggs S. Rapid diet assessment screening tools for cardiovascular disease risk reduction across healthcare settings: a scientific statement from the american heart association. Circ Cardiovasc Qual Outcomes. (2020) 13:e000094. 10.1161/HCQ.0000000000000094 32762254

[37] Sotos-Prieto M Bhupathiraju SN Mattei J Fung TT Li Y Pan A Willett WC. Association of changes in diet quality with total and cause-specific mortality. N Engl J Med. (2017) 377:143–53. 10.1056/NEJMoa1613502 28700845 PMC5589446

[38] Yang Y Dugue PA Lynch BM Hodge AM Karahalios A MacInnis RJ. Trajectories of body mass index in adulthood and all-cause and cause-specific mortality in the Melbourne Collaborative Cohort Study. BMJ Open. (2019) 9:e030078. 10.1136/bmjopen-2019-030078 31401610 PMC6701564

[39] Schroder H Cardenas-Fuentes G Martinez-Gonzalez MA Corella D Vioque J Romaguera D. Effectiveness of the physical activity intervention program in the PREDIMED-Plus study: a randomized controlled trial. Int J Behav Nutr Phys Act. (2018) 15:110. 10.1186/s12966-018-0741-x 30424822 PMC6234632

[40] Martinez-Gonzalez MA Martinez JA Hu FB Gibney MJ Kearney J. Physical inactivity, sedentary lifestyle and obesity in the European Union. Int J Obes Relat Metab Disord. (1999) 23:1192–201. 10.1038/sj.ijo.0801049 10578210

[41] Hamer M Stamatakis E Steptoe A. Effects of substituting sedentary time with physical activity on metabolic risk. Med Sci Sports Exerc. (2014) 46:1946–50. 10.1249/MSS.0000000000000317 24674977 PMC4186723

[42] Okubo H Sasaki S Rafamantanantsoa HH Ishikawa-Takata K Okazaki H Tabata I. Validation of self-reported energy intake by a self-administered diet history questionnaire using the doubly labeled water method in 140 Japanese adults. Eur J Clin Nutr. (2008) 62:1343–50. 10.1038/sj.ejcn.1602858 17671444

