# Supplementary material for: Association Between Temporal Changes in Diet Quality and Concurrent Changes in Dietary Intake, Body Mass Index, and Physical Activity Among Japanese Adults: A Longitudinal Study
Source: Front Nutr. 2022 Feb 8;9:753127. doi: 10.3389/fnut.2022.753127 (PMC8861436; doi:10.3389/fnut.2022.753127)
Supplement: Supplementary file 1 [file Data_Sheet_1.pdf]

Supporting information

"Association between temporal changes in diet quality and concurrent changes in dietary intake, body mass index, and physical activity among Japanese adults: A longitudinal study"

SUPPLEMENTARY TABLES

Table S1 Number of individuals with information on all variables by in-person testing

Table S2 Age- and sex-specific reference daily intake value according to the Dietary Reference Intakes for Japanese 2015 used for the calculation of Nutrient-Rich Food Index 9.3

Table S3 Baseline characteristics of dietary intake according to the NRF9.3 score trajectory groups

Table S4 Characteristics of the participants who were included in and excluded from this study

Table S5 Sensitivity analyses for associations of changes in the NRF9.3 score with dietary intake and physical activity using multivariate longitudinal analysis

Table S6 Associations of changes in the NR9 score with dietary intake and physical activity using multivariate longitudinal analysis

Table S7 Associations of changes in the LIM3 score with dietary intake and physical activity using multivariate longitudinal analysis

Table S8 Associations between change in the NRF9.3 score and constituent nutrients using the generalized estimating equations

Table S9 Associations of change in the NRF9.3 score with dietary intake and physical activity using the generalized estimating equations

SUPPLEMENTARY FIGURES

Figure S1 Longitudinal trajectories of the diet quality score in 211 men (1142 measurements) and 486 women (2976 measurements)

Supplementary Table S1. Number of individuals with information on all variables by in-person testing

	In-person testing (*n* = 697) [4118 measurements]												
	1	2	3	5	6	7	8	9	10	11	12	13	Total
Years of investigation	2007	2008	2009	2010	2011	2012	2013	2014	2015	2016	2017	2018	
Age	216	357	479	538	469	573	443	327	221	331	153	11	4118
Sex	216	357	479	538	469	573	443	327	221	331	153	11	4118
Area	216	357	479	538	469	573	443	327	221	331	153	11	4118
Height	216	357	479	538	469	573	443	327	221	331	153	11	4118
Body weight	216	357	479	538	469	573	443	327	221	331	153	11	4118
Body mass index	216	357	479	538	469	573	443	327	221	331	153	11	4118
Smoking status	216	357	479	538	469	573	443	327	221	331	153	11	4118
Comorbidity score [a]	205	342	478	535	465	565	371	256	219	331	151	11	3929
Step count	200	340	452	531	462	569	384	254	219	328	132	1	3872
Physical activity	195	340	452	531	462	569	384	254	219	328	132	1	3867
Dietary survey	216	357	479	538	469	573	443	327	221	331	153	11	4118

[a] Number of individuals which could obtain information on any of the 10 types of comorbidity status (including hypertension, dyslipidemia, diabetes, ischemic heart disease, other heart diseases, cerebrovascular diseases, renal failure, cancer, osteoporosis, and depression).

Supplementary Table S2. Age- and sex-specific reference daily intake value according to the Dietary Reference Intakes for Japanese 2015 used for the calculation of Nutrient-Rich Food Index 9.3

		18-29 years		30-49 years		50-69 years		≥70 years	
	Unit	Men	Women	Men	Women	Men	Women	Men	Women
Energy intake [a]	Kcal	2650	1950	2650	2000	2650	1950	2650	2000
Qualifying nutrients									
Protein [b]	g	60	50	60	50	60	50	60	50
Dietary fiber [c]	g	20	18	20	18	20	18	20	18
Vitamin A [b]	µg RAE	850	650	900	700	850	650	900	700
Vitamin C [b]	mg	100	100	100	100	100	100	100	100
Vitamin D [d]	µg	5.5	5.5	5.5	5.5	5.5	5.5	5.5	5.5
Calcium [b]	mg	800	650	650	650	800	650	650	650
Iron [b]	mg	7	10.5	7.5	10.5	7	10.5	7.5	10.5
Potassium [c]	mg	3000	2600	3000	2600	3000	2600	3000	2600
Magnesium [c]	mg	340	270	370	290	340	270	370	290
Disqualifying nutrients									
Added sugars [e]	g	33.1	24.4	33.1	25	33.1	24.4	33.1	25
Saturated fat [f]	g	20.6	15.2	20.6	15.6	20.6	15.2	20.6	15.6
Sodium [c,g]	g NaCl equivalent	8	7	8	7	8	7	8	7

RAE, retinol activity equivalent. [a] Estimated Energy Requirement (EER) for moderate level of physical activity. Energy intake conversion factor: 1 kcal=4.184 kJ. [b] Recommended Dietary Allowance (RDA). [c] Tentative dietary goal for preventing lifestyle-related diseases (DG). [d] Adequate Intake (AI). [e] Determined based on the World Health Organization's conditional recommendation (5% of energy). [f] Determined based on the DG value (7% of energy). [g] 7 g NaCl equivalent = 2756 mg sodium; 8 g NaCl equivalent = 3150 mg sodium.

Supplementary Table S3. Baseline characteristics of dietary intake according to the NRF9.3 score trajectory groups

	Total		NRF9.3 score trajectory group						
			Low-increasing		Medium-increasing		High-stable		*p*-value
N (%)	697	(100)	94	(13.5)	338	(48.5)	265	(38.0)	
**NRF9.3 score components**									
Protein [% of energy]	15.2	(2.8)	13.1	(2.5)	15.1	(2.9)	16.1	(2.3)	<0.001
Dietary fiber [g/1000 kcal]	7.5	(2.4)	5.3	(1.5)	6.9	(1.9)	8.9	(2.3)	<0.001
Vitamin A [μg RAE/1000 kcal]	465	(211)	309	(176)	450	(194)	540	(209)	<0.001
Vitamin C [mg/1000 kcal]	75	(33)	45	(22)	69	(27)	93	(33)	<0.001
Vitamin D [μg/1000 kcal]	7.3	(4.3)	5.2	(2.8)	7.4	(4.7)	8.0	(3.9)	<0.001
Calcium [mg/1000 kcal]	327	(114)	237	(99)	314	(111)	376	(98)	<0.001
Iron [mg/1000 kcal]	4.6	(1.2)	3.5	(0.9)	4.4	(1.0)	5.2	(1.0)	<0.001
Potassium [mg/1000 kcal]	1572	(431)	1135	(286)	1491	(359)	1831	(391)	<0.001
Magnesium [mg/1000 kcal]	150	(32)	119	(21)	145	(29)	168	(28)	<0.001
Added sugars [% of energy)	3.0	(1.8)	3.7	(2.4)	3.0	(1.8)	2.8	(1.5)	<0.001
Saturated fats [% of energy)	7.0	(1.8)	6.7	(2.3)	7.2	(1.9)	6.9	(1.5)	0.069
Sodium [mg/1000 kcal]	2234	(452)	2131	(413)	2303	(483)	2181	(409)	<0.001
**Foods**									
Cereals [1 g/1000 kcal]	208.2	(62.8)	236.0	(78.8)	206.0	(63.1)	201.2	(53.0)	<0.001
Pulses [1 g/1000 kcal]	36.0	(22.9)	21.7	(15.1)	32.7	(20.7)	45.3	(24.0)	<0.001
Potatoes [1 g/1000 kcal]	29.1	(21.6)	15.0	(12.2)	26.6	(18.0)	37.2	(24.8)	<0.001
Green and yellow vegetables [1 g/1000 kcal]	63.2	(38.9)	30.2	(19.0)	55.5	(32.7)	84.8	(39.5)	<0.001
Other vegetables [1 g/1000 kcal]	83.1	(46.1)	51.6	(25.4)	73.3	(34.7)	106.7	(52.9)	<0.001
Pickled vegetables [1 g/1000 kcal]	7.9	(8.5)	7.4	(8.3)	8.3	(8.4)	7.5	(8.6)	0.398
Mushrooms [1 g/1000 kcal]	7.5	(5.6)	3.9	(3.8)	6.6	(4.9)	9.8	(6.1)	<0.001
Seaweeds [1 g/1000 kcal]	8.2	(7.0)	5.7	(5.8)	7.6	(6.6)	10.0	(7.3)	<0.001
Fruits [1 g/1000 kcal]	55.5	(42.8)	32.9	(32.6)	49.1	(35.8)	71.7	(48.2)	<0.001
Fish and shellfish [1 g/1000 kcal]	42.9	(22.6)	32.4	(15.2)	43.6	(24.4)	45.9	(21.4)	<0.001
Meat [1 g/1000 kcal]	36.0	(17.2)	34.4	(23.2)	37.4	(16.7)	34.7	(15.1)	0.088
Eggs [1 g/1000 kcal]	19.4	(12.2)	17.7	(11.8)	19.0	(12.4)	20.5	(12.0)	0.119
Dairy products [1 g/1000 kcal]	78.4	(61.1)	55.7	(67.6)	74.9	(61.3)	90.9	(55.4)	<0.001
Oil [1 g/1000 kcal]	9.1	(3.8)	8.8	(3.7)	9.6	(3.8)	8.5	(3.8)	<0.001
Green tea [1 g/1000 kcal]	144.3	(131.2)	103.3	(126.6)	146.0	(133.3)	156.8	(127.7)	0.003
Black and oolong tea [1 g/1000 kcal]	76.6	(103.2)	67.1	(116.2)	69.1	(94.5)	89.6	(107.9)	0.033
Coffee [1 g/1000 kcal]	146.4	(112.8)	169.8	(116.7)	149.4	(117.5)	134.2	(103.6)	0.025
Fruit and vegetable juice [1 g/1000 kcal]	31.6	(50.8)	19.3	(39.6)	33.0	(51.9)	34.1	(52.4)	0.039
Soft drinks [1 g/1000 kcal]	26.1	(47.3)	49.9	(74.2)	25.6	(43.9)	18.3	(35.2)	<0.001
Alcoholic beverages [1 g/1000 kcal]	76.9	(126.9)	101.8	(150.1)	89.7	(142.0)	51.6	(87.8)	<0.001
Sugar and confectioneries [1 g/1000 kcal]	37.3	(23.3)	43.2	(29.8)	38.9	(23.9)	33.2	(18.9)	<0.001

NRF9.3, Nutrient-Rich Food Index 9.3. All variables with regard to dietary intake are expressed as mean and standard deviation and groups were compared using the analysis of variance.

Supplementary Table S4. Characteristics of the participants who were included in and excluded from this study

	Included participants			Excluded participants			*p*-value
	n	Distribution		*n*	Distribution		
Age [years][a]	697	52.5	(11.5)	63	40.0	(13.6)	<0.001
Women [*n* (%)][b]	697	486	(69.7)	62	36	(58.1)	0.039
Local area [*n* (%)][b]	697	232	(33.3)	63	24	(38.1)	0.439
Body mass index [kg/m^2][a]	697	22.5	(2.9)	63	22.4	(3.2)	0.994
No comorbidity [*n* (%)][b]	697	530	(76.0)	64	50.0	(79.4)	0.344
Smoker [*n* (%)][b]	697	197	(28.3)	63	17	(27.0)	0.829
Alcohol drinker [*n* (%)][b]	697	514	(73.7)	62	46	(74.2)	0.784
Energy intake [kcal/day][a]	697	1816	(491)	62	1753	(69.9)	0.341
Step count [steps/day][a]	697	10192	(3543)	60	9479	(3311)	0.120
Sedentary behavior [min/day][a]	697	220	(47)	60	226	(43)	0.263
LPA [min/day][a]	697	354	(94)	60	331	(93)	0.120
MPA [min/day][a]	697	57	(25)	60	55	(24)	0.592
VPA [min/day][a]	697	2	(7)	60	4	(11)	0.020
NR9 score[a]	697	838	(71)	62	824	(70)	0.131
LIM3 score[a]	697	73	(33)	62	81	(28)	0.071
NRF9.3 score[a]	697	765	(68)	62	743	(67)	0.015

LPA, low intensity physical activity; MPA, moderate intensity physical activity; VPA, vigorous intensity physical activity; NR9, 9 nutrients to be encouraged (protein, total dietary fibre, vitamins A, C, and D, calcium, iron, potassium, and magnesium); LIM3, 3 nutrients to be limited (added sugars, saturated fats, and sodium); NRF9.3, Nutrient-Rich Food Index 9.3

[a] Continuous variables are expressed as mean and standard deviation and groups were compared using the unpaired *t*-test.

[b] Categorical variables are expressed as number and percentage and groups were compared using the chi-square test.

Supplementary Table S5 Sensitivity analyses for associations of changes in the NRF9.3 score with dietary intake and physical activity using multivariate longitudinal analysis

Increment effects/unit	Total		NRF9.3 score trajectory group					
			Low-increasing		Medium-increasing		High-stable	
	RC	95% CI	RC	95% CI	RC	95% CI	RC	95% CI
N [measurement]	668 [3829]		90 [487]		321 [1872]		257 [1470]	
Within R^2	$R^2 = 0.318$		$R^2 = 0.696$		$R^2 = 0.392$		$R^2 = 0.380$	
Age [1 year]	0.051	(-0.174 to 0.276)	-0.483	(-1.151 to 0.185)	0.351	(0.143 to 0.559)*	0.305	(0.126 to 0.483)*
Women	1.109	(-5.463 to 7.681)	-29.661	(-46.378 to -12.943)*	-7.174	(-12.93 to -1.416)*	12.187	(6.734 to 17.640)*
Local area	-0.894	(-6.611 to 4.823)	-17.568	(-33.057 to -2.079)*	3.062	(-2.012 to 8.135)	2.911	(-1.133 to 6.955)
Body mass index [1 kg/m^2]	-1.120	(-1.896 to -0.343)*	-0.034	(-1.918 to 1.849)	-0.207	(-0.918 to 0.504)	-0.788	(-1.357 to -0.218)*
Comorbidity score [1 point]	-0.796	(-3.003 to 1.411)	-1.717	(-8.499 to 5.065)	-0.486	(-2.886 to 1.914)	-0.606	(-2.745 to 1.533)
Smoker	-4.249	(-9.316 to 0.818)	2.156	(-11.179 to 15.491)	-2.859	(-7.339 to 1.621)	0.402	(-4.119 to 4.922)
Energy intake [1 kcal]	0.017	(0.012 to 0.020)*	0.013	(0.002 to 0.022)*	0.015	(0.010 to 0.018)*	0.015	(0.010 to 0.018)*
Cereals [1 g/1000 kcal]	0.040	(-0.019 to 0.099)	0.153	(0.005 to 0.301)*	0.012	(-0.054 to 0.078)	0.042	(-0.026 to 0.110)
Pulses [1 g/1000 kcal]	0.439	(0.365 to 0.513)*	1.081	(0.825 to 1.336)*	0.330	(0.244 to 0.416)*	0.182	(0.110 to 0.253)*
Potatoes [1 g/1000 kcal]	0.494	(0.423 to 0.565)*	1.034	(0.714 to 1.353)*	0.429	(0.336 to 0.522)*	0.252	(0.186 to 0.318)*
Green and yellow vegetables [1 g/1000 kcal]	0.429	(0.380 to 0.476)*	1.230	(1.015 to 1.444)*	0.361	(0.301 to 0.420)*	0.231	(0.186 to 0.275)*
Other vegetables [1 g/1000 kcal]	0.193	(0.152 to 0.234)*	0.724	(0.553 to 0.894)*	0.189	(0.133 to 0.243)*	0.106	(0.068 to 0.142)*
Pickled vegetables [1 g/1000 kcal]	-0.231	(-0.396 to -0.066)*	0.515	(-0.015 to 1.045)	-0.070	(-0.271 to 0.131)	-0.620	(-0.782 to -0.458)*
Mushrooms [1 g/1000 kcal]	0.401	(0.111 to 0.689)*	-0.420	(-1.657 to 0.818)	0.059	(-0.298 to 0.415)	0.086	(-0.172 to 0.344)
Seaweeds [1 g/1000 kcal]	-0.066	(-0.297 to 0.164)	-0.373	(-1.142 to 0.396)	0.149	(-0.134 to 0.432)	-0.195	(-0.414 to 0.024)*
Fruits [1 g/1000 kcal]	0.287	(0.243 to 0.330)*	0.692	(0.533 to 0.850)*	0.254	(0.200 to 0.307)*	0.109	(0.068 to 0.150)*
Fish and shellfish [1 g/1000 kcal]	-0.083	(-0.168 to 0.002)	0.564	(0.299 to 0.829)*	-0.048	(-0.143 to 0.048)	-0.446	(-0.535 to -0.357)*
Meat [1 g/1000 kcal]	-0.164	(-0.270 to -0.056)*	0.185	(-0.105 to 0.476)	-0.216	(-0.337 to -0.094)*	-0.388	(-0.507 to -0.267)*
Eggs [1 g/1000 kcal]	0.198	(0.073 to 0.322)*	0.652	(0.259 to 1.044)*	0.146	(0.006 to 0.286)*	-0.036	(-0.163 to 0.091)
Dairy products [1 g/1000 kcal]	0.036	(0.004 to 0.067)*	0.224	(0.128 to 0.319)*	-0.002	(-0.037 to 0.034)	-0.022	(-0.056 to 0.012)
Oil [1 g/1000 kcal]	-0.234	(-0.673 to 0.205)	-0.376	(-1.576 to 0.823)	-0.173	(-0.684 to 0.337)	-0.664	(-1.143 to -0.183)*
Green tea [1 g/1000 kcal]	0.040	(0.028 to 0.051)*	0.060	(0.019 to 0.100)*	0.024	(0.011 to 0.037)*	0.016	(0.004 to 0.027)*
Black and oolong tea [1 g/1000 kcal]	0.010	(-0.004 to 0.025)	-0.016	(-0.061 to 0.029)	0.005	(-0.013 to 0.022)	0.003	(-0.011 to 0.017)
Coffee [1 g/1000 kcal]	0.027	(0.011 to 0.041)*	0.094	(0.052 to 0.136)*	0.027	(0.011 to 0.041)*	0.003	(-0.012 to 0.018)
Fruit and vegetable juice [1 g/1000 kcal]	0.137	(0.107 to 0.166)*	0.312	(0.222 to 0.402)*	0.140	(0.103 to 0.177)*	0.034	(0.005 to 0.062)*
Soft drinks [1 g/1000 kcal]	-0.042	(-0.075 to -0.008)*	0.042	(-0.024 to 0.108)	-0.068	(-0.113 to -0.022)*	0.004	(-0.033 to 0.042)
Alcoholic beverages [1 g/1000 kcal]	0.001	(-0.024 to 0.025)	0.041	(-0.023 to 0.105)	0.019	(-0.005 to 0.044)	-0.011	(-0.040 to 0.018)
Sugar and confectioneries [1 g/1000 kcal]	-0.268	(-0.390 to -0.144)*	-0.009	(-0.307 to 0.290)	-0.209	(-0.344 to -0.073)*	-0.359	(-0.506 to -0.211)*
Sedentary behavior [1 min/day]	0.040	(-0.203 to 0.284)	-0.116	(-0.219 to -0.013)*	0.002	(-0.033 to 0.038)	0.029	(-0.006 to 0.064)
LPA [1 min/day]	-0.045	(-0.184 to 0.094)	0.011	(-0.052 to 0.073)	-0.010	(-0.031 to 0.011)	-0.010	(-0.029 to 0.009)
MPA [1 min/day]	-0.138	(-0.652 to 0.376)	-0.159	(-0.350 to 0.033)	0.007	(-0.070 to 0.084)	-0.013	(-0.075 to 0.049)
VPA [1 min/day]	0.812	(-0.762 to 2.385)	0.649	(-0.129 to 1.426)	-0.259	(-0.545 to 0.027)	0.059	(-0.092 to 0.209)

NRF9.3, Nutrient-Rich Food Index 9.3; RC, regression coefficient; CI, confidence interval; LPA, low intensity physical activity; MPA, moderate intensity physical activity; VPA, vigorous intensity physical activity. The results of these analyses are presented as RC and 95% CI, which were calculated for each variable per unit increment. The asterisk (*) indicates statistical significance ($p<0.05$). Sex and area were time-stable variables, while the other covariates were time-varying variables.

Supplementary Table S6. Associations of changes in the NR9 score with dietary intake and physical activity using multivariate longitudinal analysis

Increment effects/unit	Total		NRF9.3 score trajectory group					
			Low-increasing		Medium-increasing		High-stable	
	RC	95% CI	RC	95% CI	RC	95% CI	RC	95% CI
N [measurement]	697 [4118]		94 [531]		338 [2007]		265 [1580]	
Within R^2	$R^2 = 0.429$		$R^2 = 0.755$		$R^2 = 0.522$		$R^2 = 0.361$	
Age [1 year]	-0.084	(-0.283 to 0.116)	-0.738	(-1.371 to -0.104)*	0.070	(-1.371 to -0.104)*	0.082	(-0.051 to 0.216)
Women	-26.990	(-32.983 to -20.996)*	-59.965	(-76.244 to -43.685)*	-33.187	(-39.291 to -27.081)*	-13.491	(-17.68 to -9.300)*
Local area	-6.095	(-11.256 to -0.933)*	-19.443	(-34.526 to -4.359)*	-3.775	(-9.146 to 1.595)	-0.623	(-3.698 to 2.451)
Body mass index [1 kg/m^2]	-0.873	(-1.575 to -0.170)*	-0.494	(-2.333 to 1.345)	-0.308	(-1.078 to 0.462)	-0.173	(-0.614 to 0.268)
Comorbidity score [1 point]	-2.117	(-4.029 to -0.204)*	-0.873	(-7.067 to 5.320)	-1.997	(-4.336 to 0.343)	-1.568	(-3.120 to -0.015)*
Smoker	-3.057	(-7.399 to 1.284)	3.697	(-8.803 to 16.198)	-0.088	(-4.760 to 4.584)	-2.457	(-5.828 to 0.913)
Energy intake [1 kcal]	0.000	(-0.002 to 0.003)	-0.001	(-0.010 to 0.008)	0.001	(-0.002 to 0.005)	0.000	(-0.002 to 0.002)
Cereals [1 g/1000 kcal]	-0.008	(-0.060 to 0.044)	0.062	(-0.075 to 0.198)	0.017	(-0.046 to 0.079)	-0.077	(-0.128 to -0.026)*
Pulses [1 g/1000 kcal]	0.422	(0.357 to 0.486)*	1.015	(0.786 to 1.243)*	0.411	(0.329 to 0.493)*	0.159	(0.106 to 0.211)*
Potatoes [1 g/1000 kcal]	0.389	(0.327 to 0.451)*	1.037	(0.753 to 1.319)*	0.429	(0.342 to 0.515)*	0.093	(0.044 to 0.141)*
Green and yellow vegetables [1 g/1000 kcal]	0.409	(0.367 to 0.450)*	1.186	(0.993 to 1.378)*	0.410	(0.354 to 0.465)*	0.208	(0.175 to 0.240)*
Other vegetables [1 g/1000 kcal]	0.174	(0.137 to 0.209)*	0.740	(0.587 to 0.891)*	0.215	(0.163 to 0.265)*	0.070	(0.043 to 0.096)*
Pickled vegetables [1 g/1000 kcal]	0.578	(0.432 to 0.722)*	1.239	(0.752 to 1.726)*	0.612	(0.423 to 0.800)*	0.233	(0.113 to 0.352)*
Mushrooms [1 g/1000 kcal]	0.541	(0.287 to 0.794)*	-0.015	(-1.107 to 1.077)	0.372	(0.035 to 0.708)*	0.353	(0.161 to 0.544)*
Seaweeds [1 g/1000 kcal]	0.188	(-0.014 to 0.390)	0.216	(-0.479 to 0.912)	0.444	(0.174 to 0.713)*	0.044	(-0.116 to 0.204)
Fruits [1 g/1000 kcal]	0.234	(0.196 to 0.272)*	0.606	(0.459 to 0.751)*	0.251	(0.200 to 0.301)*	0.050	(0.019 to 0.079)*
Fish and shellfish [1 g/1000 kcal]	0.503	(0.428 to 0.578)*	0.986	(0.747 to 1.223)*	0.535	(0.443 to 0.626)*	0.119	(0.052 to 0.184)*
Meat [1 g/1000 kcal]	0.192	(0.098 to 0.286)*	0.531	(0.259 to 0.801)*	0.157	(0.043 to 0.271)*	-0.090	(-0.177 to -0.002)*
Eggs [1 g/1000 kcal]	0.378	(0.268 to 0.488)*	0.784	(0.426 to 1.142)*	0.349	(0.215 to 0.483)*	0.068	(-0.027 to 0.163)
Dairy products [1 g/1000 kcal]	0.146	(0.118 to 0.174)*	0.334	(0.244 to 0.422)*	0.124	(0.090 to 0.158)*	0.054	(0.027 to 0.079)*
Oil [1 g/1000 kcal]	0.565	(0.179 to 0.951)*	-0.059	(-1.174 to 1.056)	0.800	(0.318 to 1.281)*	0.069	(-0.284 to 0.422)
Green tea [1 g/1000 kcal]	0.038	(0.027 to 0.048)*	0.074	(0.036 to 0.110)*	0.030	(0.016 to 0.042)*	0.008	(0.000 to 0.017)*
Black and oolong tea [1 g/1000 kcal]	0.017	(0.004 to 0.030)*	0.004	(-0.037 to 0.045)	0.014	(-0.002 to 0.030)	0.007	(-0.003 to 0.017)
Coffee [1 g/1000 kcal]	0.032	(0.018 to 0.044)*	0.090	(0.052 to 0.128)*	0.033	(0.017 to 0.048)*	0.005	(-0.006 to 0.016)
Fruit and vegetable juice [1 g/1000 kcal]	0.128	(0.101 to 0.153)*	0.310	(0.225 to 0.393)*	0.151	(0.116 to 0.186)*	0.010	(-0.011 to 0.030)
Soft drinks [1 g/1000 kcal]	-0.070	(-0.098 to -0.040)*	-0.016	(-0.077 to 0.045)	-0.061	(-0.103 to -0.018)*	-0.046	(-0.072 to -0.018)*
Alcoholic beverages [1 g/1000 kcal]	-0.036	(-0.057 to -0.013)*	-0.001	(-0.060 to 0.057)	-0.007	(-0.031 to 0.017)	-0.061	(-0.083 to -0.039)*
Sugar and confectioneries [1 g/1000 kcal]	0.283	(0.175 to 0.390)*	0.556	(0.279 to 0.833)*	0.370	(0.242 to 0.497)*	-0.045	(-0.154 to 0.063)
Sedentary behavior [1 min/day]	-0.016	(-0.048 to 0.016)	-0.157	(-0.251 to -0.061)*	-0.005	(-0.042 to 0.031)	0.020	(-0.006 to 0.047)
LPA [1 min/day]	-0.010	(-0.028 to 0.008)	0.002	(-0.055 to 0.060)	-0.005	(-0.027 to 0.016)	-0.010	(-0.024 to 0.004)
MPA [1 min/day]	0.014	(-0.047 to 0.076)	-0.163	(-0.343 to 0.017)	0.049	(-0.028 to 0.126)	0.023	(-0.024 to 0.070)
VPA [1 min/day]	-0.074	(-0.265 to 0.116)	0.446	(-0.289 to 1.182)	-0.379	(-0.659 to -0.099)*	-0.082	(-0.196 to 0.032)

NRF9.3, Nutrient-Rich Food Index 9.3; RC, regression coefficient; CI, confidence interval; LPA, low intensity physical activity; MPA, moderate intensity physical activity; VPA, vigorous intensity physical activity. The results of these analyses are presented as RC and 95% CI, which were calculated for each variable per unit increment. The asterisk (*) indicates statistical significance (p<0.05). Sex and area were time-stable variables, while the other covariates were time-varying variables.

Supplementary Table S7. Associations of changes in the LIM3 score with dietary intake and physical activity using multivariate longitudinal analysis

Increment effects/unit	Total		NRF9.3 score trajectory group					
			Low-increasing		Medium-increasing		High-stable	
	RC	95% CI	RC	95% CI	RC	95% CI	RC	95% CI
N [measurement]	697 [4118]		94 [531]		338 [2007]		265 [1580]	
Within R^2	$R^2 = 0.464$		$R^2 = 0.476$		$R^2 = 0.462$		$R^2 = 0.498$	
Age [1 year]	-0.139	(-0.248 to -0.029)*	-0.427	(-0.824 to -0.030)*	-0.156	(-0.307 to -0.004)*	0.199	(-0.349 to -0.047)*
Women	-27.988	(-31.246 to -24.729)*	-31.086	(-41.517 to -20.655)*	-25.636	(-29.982 to -21.290)*	-26.068	(-30.923 to -21.212)*
Local area	-5.347	(-8.135 to -2.559)*	-5.178	(-14.998 to 4.642)	-6.680	(-10.607 to -2.752)*	-3.599	(-7.237 to 0.038)
Body mass index [1 kg/m^2]	0.242	(-0.140 to 0.624)	-0.547	(-1.718 to 0.625)	0.111	(-0.439 to 0.661)	0.670	(0.150 to 1.189)*
Comorbidity score [1 point]	-1.448	(-2.518 to -0.377)	-0.101	(-3.690 to 3.487)	-2.060	(-3.565 to -0.554)*	-1.000	(-2.593 to 0.593)
Smoker	1.436	(-0.959 to 3.831)	5.706	(-2.037 to 13.448)	1.990	(-1.222 to 5.202)	-2.835	(-6.510 to 0.840)
Energy intake [1 kcal]	-0.016	(-0.017 to -0.014)*	-0.015	(-0.020 to -0.009)*	-0.015	(-0.017 to -0.012)*	-0.015	(-0.017 to -0.011)*
Cereals [1 g/1000 kcal]	-0.064	(-0.092 to -0.034)*	-0.061	(-0.140 to 0.018)	-0.033	(-0.073 to 0.007)	-0.131	(-0.181 to -0.079)*
Pulses [1 g/1000 kcal]	-0.019	(-0.055 to 0.016)	-0.066	(-0.196 to 0.064)	0.022	(-0.029 to 0.074)	-0.033	(-0.085 to 0.019)
Potatoes [1 g/1000 kcal]	-0.110	(-0.145 to -0.075)*	0.015	(-0.145 to 0.176)	-0.031	(-0.085 to 0.022)	-0.172	(-0.219 to -0.124)*
Green and yellow vegetables [1 g/1000 kcal]	-0.015	(-0.038 to 0.008)	-0.016	(-0.126 to 0.093)	0.000	(-0.034 to 0.034)	-0.002	(-0.034 to 0.030)
Other vegetables [1 g/1000 kcal]	-0.019	(-0.039 to 0.000)*	-0.026	(-0.113 to 0.060)	0.010	(-0.022 to 0.041)	-0.037	(-0.063 to -0.010)*
Pickled vegetables [1 g/1000 kcal]	0.791	(0.709 to 0.872)*	0.704	(0.427 to 0.980)*	0.706	(0.587 to 0.824)*	0.815	(0.696 to 0.932)*
Mushrooms [1 g/1000 kcal]	0.110	(-0.032 to 0.252)	0.528	(-0.096 to 1.151)	0.218	(0.007 to 0.429)*	0.117	(-0.074 to 0.308)
Seaweeds [1 g/1000 kcal]	0.298	(0.184 to 0.412)*	0.594	(0.194 to 0.992)*	0.260	(0.089 to 0.429)*	0.261	(0.103 to 0.418)*
Fruits [1 g/1000 kcal]	-0.049	(-0.070 to -0.027)*	-0.034	(-0.117 to 0.050)	-0.025	(-0.057 to 0.006)	-0.060	(-0.090 to -0.029)*
Fish and shellfish [1 g/1000 kcal]	0.574	(0.531 to 0.615)*	0.547	(0.410 to 0.682)*	0.561	(0.502 to 0.619)*	0.557	(0.490 to 0.623)*
Meat [1 g/1000 kcal]	0.332	(0.278 to 0.384)*	0.315	(0.156 to 0.473)*	0.335	(0.263 to 0.406)*	0.300	(0.211 to 0.387)*
Eggs [1 g/1000 kcal]	0.160	(0.098 to 0.221)*	0.113	(-0.094 to 0.320)	0.189	(0.104 to 0.274)*	0.108	(0.011 to 0.204)*
Dairy products [1 g/1000 kcal]	0.099	(0.083 to 0.114)*	0.117	(0.065 to 0.168)*	0.115	(0.093 to 0.136)*	0.065	(0.039 to 0.090)*
Oil [1 g/1000 kcal]	0.757	(0.540 to 0.973)*	0.534	(-0.107 to 1.175)	0.876	(0.573 to 1.179)*	0.523	(0.170 to 0.875)*
Green tea [1 g/1000 kcal]	-0.001	(-0.006 to 0.005)	0.017	(-0.004 to 0.037)	0.002	(-0.006 to 0.009)	-0.005	(-0.013 to 0.004)
Black and oolong tea [1 g/1000 kcal]	0.008	(0.000 to 0.015)*	0.007	(-0.016 to 0.031)	0.011	(0.000 to 0.021)*	0.005	(-0.005 to 0.015)
Coffee [1 g/1000 kcal]	0.009	(0.002 to 0.016)*	0.014	(-0.007 to 0.036)	0.005	(-0.005 to 0.014)	0.009	(-0.002 to 0.021)
Fruit and vegetable juice [1 g/1000 kcal]	-0.014	(-0.028 to 0.001)	-0.004	(-0.051 to 0.043)	-0.006	(-0.027 to 0.016)	-0.028	(-0.049 to -0.007)*
Soft drinks [1 g/1000 kcal]	-0.035	(-0.050 to -0.018)*	-0.057	(-0.092 to -0.021)*	-0.004	(-0.030 to 0.022)	-0.059	(-0.085 to -0.031)*
Alcoholic beverages [1 g/1000 kcal]	-0.034	(-0.046 to -0.021)*	-0.032	(-0.066 to 0.002)	-0.028	(-0.043 to -0.011)*	-0.055	(-0.077 to -0.032)*
Sugar and confectioneries [1 g/1000 kcal]	0.527	(0.466 to 0.587)*	0.601	(0.440 to 0.761)*	0.570	(0.489 to 0.651)*	0.287	(0.177 to 0.396)*
Sedentary behavior [1 min/day]	-0.014	(-0.032 to 0.003)	-0.041	(-0.096 to 0.015)	-0.005	(-0.029 to 0.019)	-0.008	(-0.036 to 0.020)
LPA [1 min/day]	0.000	(-0.010 to 0.010)	0.004	(-0.030 to 0.038)	0.001	(-0.013 to 0.015)	-0.003	(-0.017 to 0.012)
MPA [1 min/day]	0.023	(-0.011 to 0.057)	0.009	(-0.097 to 0.115)	0.035	(-0.016 to 0.086)	0.036	(-0.013 to 0.085)
VPA [1 min/day]	-0.137	(-0.242 to -0.030)*	-0.297	(-0.723 to 0.129)	-0.047	(-0.225 to 0.132)	-0.146	(-0.270 to -0.020)*

NRF9.3, Nutrient-Rich Food Index 9.3; RC, regression coefficient; CI, confidence interval; BMI, body mass index; LPA, low intensity physical activity; MPA, moderate intensity physical activity; VPA, vigorous intensity physical activity. The results of these analyses are presented as RC and 95% CI, which were calculated for each variable per unit increment. The asterisk (*) indicates statistical significance ($p<0.05$). Sex and area were time-stable variables, while the other covariates were time-varying variables.

Supplementary Table S8. Associations between change in the NRF9.3 score and constituent nutrients using the generalized estimating equations

	Total		NRF 9.3 score Trajectory Group					
			Low-increasing		Medium-increasing		High-stable	
Increment effects/unit	RC	95% CI	RC	95% CI	RC	95% CI	RC	95% CI
N	697		94		338		265	
Between R^2	$R^2 = 0.615$		$R^2 = 0.628$		$R^2 = 0.540$		$R^2 = 0.607$	
Age [1 year]	-0.017	(-0.362 to 0.327)	0.741	(-0.581 to 2.064)	0.177	(-0.220 to 0.575)	0.318	(0.014 to 0.621)*
Women	-6.806	(-11.446 to -2.165)*	-25.250	(-39.756 to -10.742)*	-7.126	(-12.294 to -1.956)*	-0.627	(-5.081 to 3.827)
Local area	-1.137	(-4.945 to 2.672)	-0.320	(-12.386 to 11.745)	-3.359	(-7.810 to 1.092)	-1.720	(-4.987 to 1.546)
Body mass index [1 kg/m^2]	-0.072	(-1.244 to 1.100)	-1.064	(-4.255 to 2.127)	0.294	(-1.142 to 1.729)	0.001	(-1.030 to 1.032)
Comorbidity score [1 point]	-0.702	(-6.558 to 5.154)	20.565	(-1.018 to 42.148)	-1.167	(-7.682 to 5.349)	-0.548	(-5.869 to 4.773)
Smoker	-1.730	(-5.824 to 2.364)	-11.651	(-23.691 to 0.390)	1.588	(-2.947 to 6.123)	-3.558	(-7.540 to 0.425)
Energy intake [1 kcal]	0.005	(-0.002 to 0.012)	0.009	(-0.012 to 0.030)	0.005	(-0.003 to 0.013)	0.001	(-0.006 to 0.008)
Protein [1 % energy]	0.966	(-1.444 to 3.376)	-5.424	(-13.465 to 2.618)	2.059	(-0.833 to 4.952)	1.258	(-1.312 to 3.828)
Dietary fibre [1 g/1000 kcal]	10.569	(6.917 to 14.22)*	25.147	(9.373 to 40.921)*	11.430	(6.693 to 16.166)*	6.328	(3.269 to 9.386)*
Vitamin A [1 µg RAE/1000 kcal]	0.027	(0.010 to 0.043)*	0.015	(-0.037 to 0.066)	0.049	(0.027 to 0.071)*	0.009	(-0.004 to 0.021)
Vitamin C [1 mg/1000 kcal]	-0.059	(-0.343 to 0.225)	0.045	(-1.086 to 1.175)	-0.352	(-0.689 to -0.013)*	0.060	(-0.212 to 0.332)
Vitamin D [1 µg/1000 kcal]	1.062	(-0.344 to 2.468)	3.916	(-1.340 to 9.172)	0.254	(-1.357 to 1.865)	0.481	(-0.763 to 1.725)
Calcium [1 mg/1000 kcal]	0.000	(-0.064 to 0.065)	-0.046	(-0.276 to 0.184)	-0.029	(-0.103 to 0.046)	-0.008	(-0.065 to 0.049)
Iron [1 mg/1000 kcal]	12.073	(4.778 to 19.368)*	10.848	(-19.033 to 40.729)	4.184	(-4.790 to 13.157)	7.229	(0.997 to 13.461)*
Potassium [1 mg/1000 kcal]	0.053	(0.030 to 0.076)*	0.073	(-0.005 to 0.150)	0.061	(0.034 to 0.087)*	0.011	(-0.010 to 0.031)
Magnesium [1 mg/1000 kcal]	-0.020	(-0.127 to 0.087)	0.151	(-0.174 to 0.476)	-0.025	(-0.141 to 0.090)	-0.051	(-0.176 to 0.074)
Added sugars [1 % energy]	-1.710	(-3.429 to 0.008)	-3.070	(-6.469 to 0.328)	-0.431	(-2.667 to 1.805)	-0.642	(-2.709 to 1.424)
Saturated fats [1 % energy]	-1.836	(-4.539 to 0.868)	6.204	(-3.211 to 15.619)	-3.440	(-6.465 to -0.413)*	-3.208	(-5.834 to -0.581)*
Sodium [1 mg/1000 kcal]	-0.048	(-0.058 to -0.037)*	-0.039	(-0.072 to -0.004)*	-0.035	(-0.047 to -0.022)*	-0.055	(-0.065 to -0.045)*

NRF9.3, Nutrient-Rich Food Index 9.3; RC, regression coefficient; CI, confidence interval. The results of these analyses are presented as RC and 95% CI, which were calculated for each variable per unit increment. The asterisk (*) indicates statistical significance ($p<0.05$). Sex and area were time-stable variables, while the other covariates were time-varying variables.

Supplementary Table S9. Associations of change in the NRF9.3 score with dietary intake and physical activity using the generalized estimating equations

Increment effects/unit	Total		NRF 9.3 score Trajectory Group					
			Low-increasing		Medium-increasing		High-stable	
	RC	95% CI	RC	95% CI	RC	95% CI	RC	95% CI
N	697		94		338		265	
Between R^2	$R^2 = 0.590$		$R^2 = 0.721$		$R^2 = 0.495$		$R^2 = 0.507$	
Age [1 year]	-0.024	(-0.406 to 0.358)	0.351	(-1.156 to 1.859)	0.266	(-0.204 to 0.735)	0.183	(-0.208 to 0.574)
Women	-4.203	(-9.421 to 1.015)	-14.290	(-30.808 to 2.228)	-7.392	(-13.477 to -1.305)*	5.045	(-0.478 to 10.567)
Local area	-1.416	(-5.616 to 2.783)	10.043	(-2.933 to 23.018)	-3.979	(-9.234 to 1.276)	-4.365	(-8.327 to -0.402)*
Body mass index [1 kg/m^2]	-0.236	(-1.481 to 1.008)	-0.603	(-4.036 to 2.830)	-0.062	(-1.630 to 1.506)	-0.063	(-1.286 to 1.161)
Comorbidity score [1 point]	0.008	(-6.258 to 6.273)	-2.716	(-24.953 to 19.521)	-0.192	(-7.236 to 6.852)	1.122	(-5.503 to 7.747)
Smoker	-0.649	(-5.064 to 3.766)	-0.379	(-12.728 to 13.486)	-0.030	(-4.991 to 4.932)	-2.860	(-7.653 to 1.933)
Energy intake [1 kcal]	0.011	(0.003 to 0.019)*	0.024	(0.000 to 0.047)*	0.007	(-0.001 to 0.016)	0.014	(0.004 to 0.023)*
Cereals [1 g/1000 kcal]	0.157	(0.021 to 0.291)*	0.487	(0.111 to 0.861)*	0.092	(-0.059 to 0.243)	-0.046	(-0.212 to 0.121)
Pulses [1 g/1000 kcal]	0.654	(0.475 to 0.832)*	2.003	(1.049 to 2.955)*	0.476	(0.254 to 0.697)*	0.173	(0.008 to 0.337)*
Potatoes [1 g/1000 kcal]	0.684	(0.493 to 0.874)*	1.574	(0.621 to 2.526)*	0.585	(0.322 to 0.847)*	0.269	(0.105 to 0.432)*
Green and yellow vegetables [1 g/1000 kcal]	0.528	(0.402 to 0.652)*	1.405	(0.662 to 2.147)*	0.443	(0.281 to 0.605)*	0.257	(0.144 to 0.369)*
Other vegetables [1 g/1000 kcal]	0.251	(0.144 to 0.357)*	1.256	(0.641 to 1.871)*	0.376	(0.218 to 0.534)*	0.062	(-0.026 to 0.150)
Pickled vegetables [1 g/1000 kcal]	-0.337	(-0.769 to 0.094)	-0.110	(-1.687 to 1.467)	-0.063	(-0.592 to 0.467)	-0.650	(-1.080 to -0.218)*
Mushrooms [1 g/1000 kcal]	0.821	(0.058 to 1.584)*	-4.236	(-7.970 to -0.502)*	-0.064	(-1.051 to 0.923)	0.333	(-0.320 to 0.986)
Seaweeds [1 g/1000 kcal]	-0.466	(-1.038 to 0.106)	-2.322	(-4.431 to -0.211)*	-0.065	(-0.797 to 0.667)	-0.358	(-0.874 to 0.159)
Fruits [1 g/1000 kcal]	0.349	(0.246 to 0.452)*	0.623	(0.107 to 1.137)*	0.260	(0.123 to 0.396)*	0.100	(0.001 to 0.197)*
Fish and shellfish [1 g/1000 kcal]	0.144	(-0.062 to 0.351)	1.385	(0.475 to 2.294)*	0.088	(-0.143 to 0.320)	-0.425	(-0.654 to -0.196)*
Meat [1 g/1000 kcal]	0.064	(-0.201 to 0.329)	0.121	(-0.562 to 0.804)	-0.078	(-0.399 to 0.244)	-0.302	(-0.619 to 0.015)
Eggs [1 g/1000 kcal]	0.353	(0.050 to 0.655)*	0.829	(-0.202 to 1.859)	0.314	(-0.032 to 0.659)	-0.223	(-0.546 to 0.100)
Dairy products [1 g/1000 kcal]	0.142	(0.064 to 0.219)*	0.426	(0.197 to 0.654)*	0.056	(-0.033 to 0.146)	-0.037	(-0.126 to 0.053)
Oil [1 g/1000 kcal]	0.014	(-1.101 to 1.130)	1.736	(-2.095 to 5.567)	0.375	(-0.933 to 1.683)	-1.730	(-2.963 to -0.497)*
Green tea [1 g/1000 kcal]	0.013	(-0.015 to 0.042)	0.050	(-0.056 to 0.156)	-0.021	(-0.055 to 0.013)	0.013	(-0.015 to 0.042)
Black and oolong tea [1 g/1000 kcal]	0.032	(-0.001 to 0.066)	0.034	(-0.076 to 0.144)	0.032	(-0.012 to 0.075)	0.029	(-0.004 to 0.061)
Coffee [1 g/1000 kcal]	0.022	(-0.010 to 0.053)	0.081	(-0.012 to 0.173)	0.034	(-0.001 to 0.069)	-0.006	(-0.042 to 0.031)
Fruit and vegetable juice [1 g/1000 kcal]	0.155	(0.081 to 0.228)*	-0.025	(-0.351 to 0.301)	0.134	(0.045 to 0.222)*	0.002	(-0.069 to 0.073)
Soft drinks [1 g/1000 kcal]	-0.170	(-0.252 to -0.087)*	0.081	(-0.115 to 0.277)	-0.100	(-0.199 to -0.001)*	-0.161	(-0.271 to -0.049)*
Alcoholic beverages [1 g/1000 kcal]	0.046	(-0.007 to 0.099)	0.167	(0.009 to 0.325)*	0.048	(-0.009 to 0.104)	-0.062	(-0.130 to 0.007)
Sugar and confectioneries [1 g/1000 kcal]	0.147	(-0.138 to 0.432)	1.051	(0.297 to 1.803)*	0.069	(-0.242 to 0.380)	-0.338	(-0.715 to 0.040)
Sedentary behavior [1 min/day]	-0.690	(-1.243 to -0.136)*	-2.029	(-4.485 to 0.427)	-0.183	(-0.940 to 0.575)	-0.291	(-0.774 to 0.192)
LPA [1 min/day]	-0.684	(-1.234 to -0.132)*	-2.030	(-4.492 to 0.432)	-0.177	(-0.931 to 0.577)	-0.307	(-0.788 to 0.174)
MPA [1 min/day]	-0.673	(-1.287 to -0.057)*	-2.589	(-5.284 to 0.106)	-0.156	(-0.985 to 0.673)	-0.210	(-0.753 to 0.332)
VPA [1 min/day]	0.000	(0.000 to 0.000)	0.000	(0.000 to 0.000)	0.000	(0.000 to 0.000)	0.000	(0.000 to 0.000)

NRF9.3, Nutrient-Rich Food Index 9.3; RC, regression coefficient; CI, confidence interval; LPA, low intensity physical activity; MPA, moderate intensity physical activity; VPA, vigorous intensity physical activity. The results of these analyses are presented as RC and 95% CI, which were calculated for each variable per unit increment. The asterisk (*) indicates statistical significance (p<0.05).

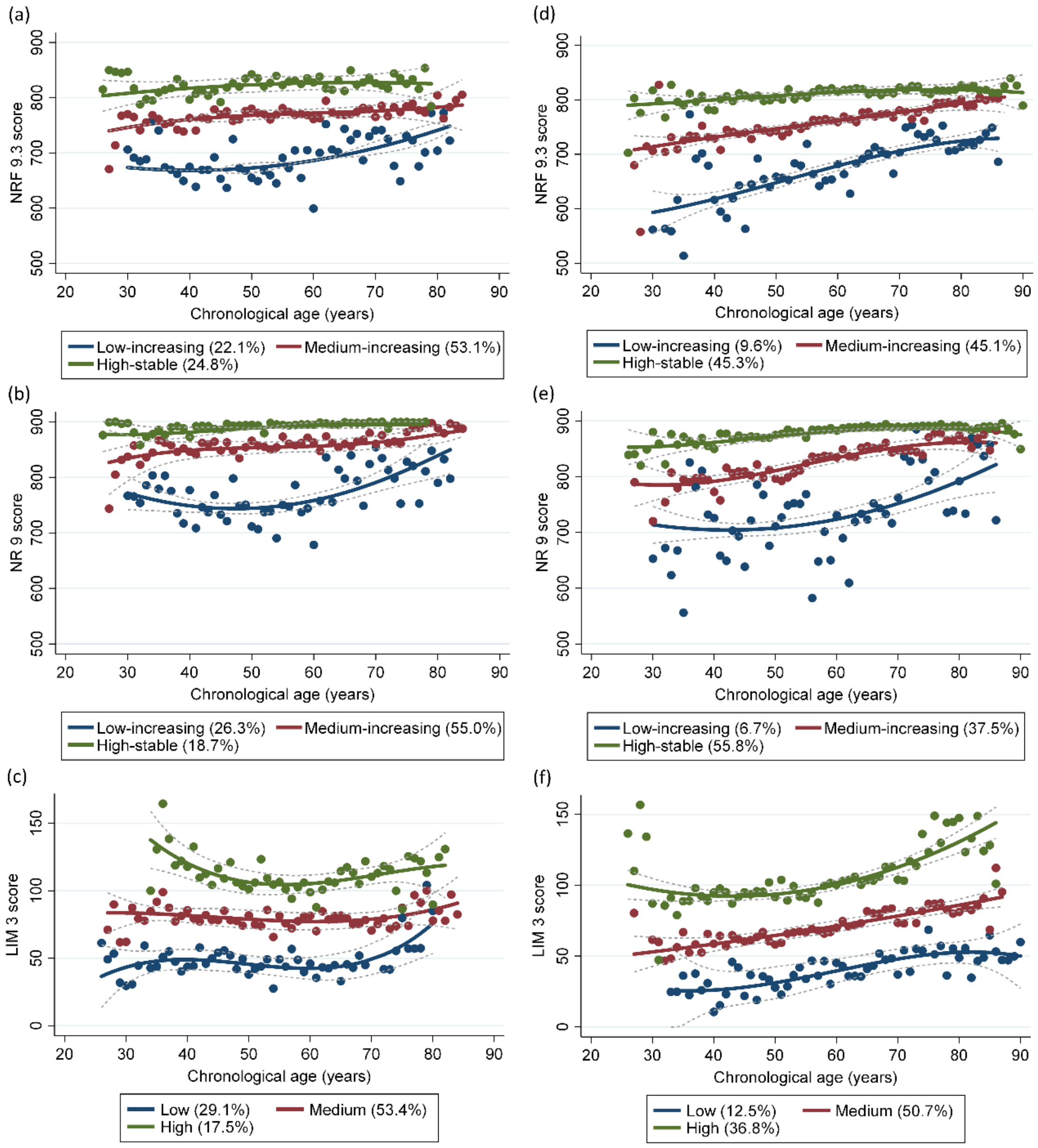

Supplementary Figure S1. Longitudinal trajectories of the diet quality score in 211 men (1142 measurements) and 486 women (2976 measurements)

The latent class growth models identified three distinct trajectory groups according to the Nutrient-Rich Food Index 9.3 (NRF9.3) score ([a] in men, [b] in women), nutrient rich 9 (NR9) score ([c] in men, [d] in women), and limiting nutrient 3 (LIM3) score ([e] in men, [f] in women) in individuals aged 26–90 years using the maximum likelihood method. Solid lines represent the group's mean diet quality score trajectory, and dashed lines represent 95% confidence intervals.
